# Supplementary material for: Sequencing-based fine-mapping and in silico functional characterization of the 10q24.32 arsenic metabolism efficiency locus across multiple arsenic-exposed populations
Source: PLoS Genet. 2023 Jan 20;19(1):e1010588. doi: 10.1371/journal.pgen.1010588 (PMC9891528; doi:10.1371/journal.pgen.1010588)
Supplement: S1 Text — a. Supplemental Methods: i. The Genotype-Tissue Expression Project. ii. Measurement of Urinary Arsenic Metabolites. iii. Quality Control for GTEx Whole Genome Sequencing Data. iv. Genotype Quality Control and Imputation. v. GTEx Expression Data Quantification. vi. Read Alignment and Genetic Variant Calling. vii. MANTRA meta-analysis. (PDF) [file pgen.1010588.s001.pdf]

SUPPLEMENTAL METHODS

The Genotype-Tissue Expression Project (GTEx)

The Genotype-Tissue Expression (GTEx) Project [1,2] was established in 2010 by the NIH to create a centralized data source for inherited genetic variation and gene expression in multiple human tissue types. The protocols for donor enrollment and the process for obtaining informed consent as well as procedures for specimen collection, sample preparation and storage, and the histological review processes for GTEx project samples have been described in previous work from the consortium. The project collects multiple tissue types from each participating donor to enable the study of tissue-specific gene expression and its relationship with genetic variation.

The protocols for data preparation have been previously described by the GTEx consortium. Briefly, genotype data was generated from whole genome sequencing of 899 samples using Illumina's HiSeq X (for all but the first sample batch which used HiSeq 2000). Samples underwent quality control filtering, resulting in a final sample of 838 individuals. Variants were jointly called using GATK HaplotypeCaller v3.5 and underwent quality control filtering prior to inclusion in the final dataset. Expression data for the project was collected through RNA sequencing with Illumina's TruSeq library construction protocol.

Measurement of Urinary Arsenic Metabolites

Separation of arsenic species in urine samples from all three populations was performed using high-performance liquid chromatography [3–5]. This procedure was followed by IPC-MS to quantify arsenic species in urine samples. Details regarding the measurement protocols and the limit of detection (LOD) for each metabolite used by each study have been described previously [4–7]. In brief, the LOD for HEALS was 1μg/L for all inorganic arsenic species measured. In order to minimize undetectable levels of iAs^{III} and iAs^{V} due to low exposure in SHS, iAs^{III} was oxidized for iAs^{V} following the procedure previously described by Scheer et al [8]. In this population, the LOD was 0.1μg/L for iAs^{V} and 0.5μg/L for DMA and MMA. Finally, the LOD for arsenic species in NHSCS were 0.15μg/L for iAs^{III}, 0.1 μg/L for iAs^{V}, 0.14 μg/L for MMA, and 0.11 μg/L for DMA.

For this analysis, DMA% was used as the primary measure of AME. In both SHS and NHSCS, measures of AME were not computed if two or more species were undetectable in an individual. In the case of one missing, metabolite, the missing value was estimated as the LOD divided by the square root of two and used in downstream analyses. Among the HEALS participants, 523, 155, and 4 participants had values below the LOD for iAsIII, iAsV, and MMA, respectively. We kept these participants in our analyses and set arsenic species values that were <LOD to zero. In SHS, 6 participants with more than two undetectable arsenic species were excluded. Additionally, imputed values for iAsV and MMA in 49 and 2 participants, respectively were calculated for SHS. In NHSCS, 39 participants with more than two missing arsenic species were excluded. We then calculate imputed values for iAsIII, iAsV, and MMA in 225, 598, and 29 participants, respectively.

Quality Control for GTEx Whole Genome Sequencing Data

Whole genome sequencing data from GTEx v8 participants were used to identify eQTLs that were used for co-localization analysis. The protocols for sequencing, processing, and quality control have been previously described by the GTEx consortium [9,10]. Briefly, sequencing was performed for 899 samples using Illumina HiSeq 2000 and Illumina HiSeq X. Samples were excluded for any of the following: low quality replicates, large chromosomal abnormalities, alignment with a different pipeline during variant calling, sepsis, cerebral palsy, relationship with another donor, and sex mismatch. This resulted in a

final sample of 838 individuals. Variants were jointly called using GATK HaplotypeCaller v3.5 and underwent quality control filtering based on the following: failed VQSR at sensitivity <99.8% for SNPs or <99.95% for indels; located in a Low Complexity Region; inbreeding coefficient <-0.3; monomorphic after the following genotype calls were set to missing: compound HET sites after splitting multi-allelic sites to bi-allelic, genotype quality <20, calls with allelic imbalance >0.8 or <0.2, or heterozygous calls in chrX nonPAR regions in males; missingness ≥15%; failed Hardy-Weinberg Equilibrium testing ($P<10^{-8}$) in European or African American subsets; significant association with sequencing technology, library batch, or PCR library preparation ($P<10^{-8}$); or significant non-random missingness of reference alleles with MAF>1%.

Genotype Quality Control and Imputation

We removed SNPs with a genotyping rate ≤80% (4,106 in HEALS, 4,612 in SHS, 4,389 in NHSCS), SNPs out of Hardy-Weinberg equilibrium P≤1x10-10 (96 in HEALS, 27 in SHS, 37 in NHSCS), SNPs with MAF≤0.005 (5,201 in HEALS, 4,770 in SHS, 4,995 in NHSCS), and samples with >50% missingness (66 in HEALS, 316 in SHS, 104 in NHSCS). An additional 13 samples were removed from HEALS due to a lack of genome-wide SNP data needed to generate a kinship matrix and control for sample relateness. These procedures resulted in 455 variants in 2,357 samples in HEALS, 449 variants in 558 individuals in SHS, and 437 variants in 648 individuals in NHSCS. We imputed missing genotype data for each of our three cohorts using the TopMed Imputation Server and the TopMed reference panel [11]. Following QC, unphased SNP data were uploaded to the server without identifiers. Following imputation, samples with low imputation quality ($R^2<0.3$) were excluded resulting in 19,761 10q24.32 variants in HEALS, 14,248 in SHS, and 21,332 in NHSCS.

GTEx Expression Data Quantification

Expression data for the Genotype-Tissue Expression (GTEx) project version 8 was produced with RNA-seq data that was obtained with the Illumina TruSeq library construction protocol [9,10]. The protocols for processing the RNA and quantifying expression have been described previously by the GTEx project [1,2,10,12]. In summary, high quality RNA samples were used for library preparation, quantified, normalization, and sequenced with HiSeq 2000 or HiSeq 2500 following the manufacturer’s protocols. RNA-seq alignment to hg38 reference was performed with STAR v2.5.3a based on GENCODE v26 annotation (https://github.com/broadinstitute/gtex-pipeline/tree/master/rnaseq) and all samples underwent quality control filtering. Gene-level expression quantification was performed by GENCODE v26 which resulted in gene expression values for all samples from a given tissue which were normalized both within tissues and between samples [13] prior to eQTL analysis. The version 8 expression data used in these analyses were downloaded from the GTEx portal (https://gtexportal.org/home/datasets).

Read Alignment and Genetic Variant Calling

Targeting sequencing data from all three cohorts (HEALS, SHS, and NH) were processed at the University of Chicago Bioinformatics Core using the Genome Analysis Toolkit (GATK) [14] Best Practices Workflow for germline short variant discovery [15]. For variant calling, raw paired-end reads were mapped to the hg19 reference using the Novoalign software. Variants were called for each sample using GATK HaplotypeCaller

in GVCF mode to produce intermediate GVCFs which were then consolidated to a single GVCF file with the GenomicsDBImport tool. We used this consolidated file to perform joint variant calling across all samples by cohort, using GATK GenotypeGVCFs, which provided a set of raw SNPs and indels in VCF format. Biallelic SNPs with any of the following properties were excluded: QualbyDepth (QD) <2.0, FisherStrand (FS) >60.0, RMSMappingQuality (MQ) <40.0, StrandOddsRatio (SOR) >3.0, MappingQualityRankSumTest (MQRankSum) <-12.5, or ReadPosRankSum <-8.0. Additionally, indels with one or more of the following properties were excluded: QD <2.0, FS >200.0, ReadPosRankSum <-20, Inbreeding Coefficient <-0.8, or SOR >10.0. Finally, samples with low coverage, indicated by Depth of Coverage (DP) <30 were removed.

MANTRA meta-analysis:

MANTRA [16,17] creates clusters of subjects with similar ancestry, assuming those within a cluster have the same allelic effects, while allowing heterogeneity between clusters. MANTRA applies a Bayesian approach to estimate the marginal likelihood under both the null and alternative for each SNP and provides a Bayes factor for each SNP which can be used to calculate posterior probabilities of association and credible sets with a 95 percent likelihood of containing the causal variant.

REFERENCES

1. Carithers LJ, Ardlie K, Barcus M, Branton PA, Britton A, Buia SA, et al. A Novel Approach to High-Quality Postmortem Tissue Procurement: The GTEx Project. Biopreserv Biobank. 2015 Oct;13(5):311–9.

2. The Genotype-Tissue Expression (GTEx) project. Nat Genet. 2013 Jun;45(6):580–5.

3. Moon K, Guallar E, Navas-Acien A. Arsenic Exposure and Cardiovascular Disease:An Updated Systematic Review. Curr Atheroscler Rep. 2012 Dec 1;14(6):542–55.

4. Ahsan H, Chen Y, Parvez F, Argos M, Hussain AI, Momotaj H, et al. Health Effects of Arsenic Longitudinal Study (HEALS): description of a multidisciplinary epidemiologic investigation. J Expo Sci Environ Epidemiol. 2006 Mar;16(2):191–205.

5. Gilbert-Diamond Diane, Li Zhigang, Perry Ann E., Spencer Steven K., Gandolfi A. Jay, Karagas Margaret R. A Population-based Case–Control Study of Urinary Arsenic Species and Squamous Cell Carcinoma in New Hampshire, USA. Environmental Health Perspectives. 2013 Oct 1;121(10):1154–60.

6. Navas -Acien Ana, Umans JG, Howard BV, Goessler W, Francesconi KA, Crainiceanu CM, et al. Urine Arsenic Concentrations and Species Excretion Patterns in American Indian Communities Over a 10-year Period: The Strong Heart Study. Environmental Health Perspectives. 2009 Sep 1;117(9):1428–33.

7. Delgado DA, Chernoff M, Huang L, Tong L, Chen L, Jasmine F, et al. Rare, Protein-Altering Variants in AS3MT and Arsenic Metabolism Efficiency: A Multi-Population Association Study. Environmental Health Perspectives. 129(4):047007.

8. Scheer J, Findenig S, Goessler W, Francesconi KA, Howard B, Umans JG, et al. Arsenic species and selected metals in human urine: validation of HPLC/ICPMS and ICPMS procedures for a long-term population-based epidemiological study. Anal Methods. 2012 Feb 2;4(2):406–13.

9. GTEx Portal [Internet]. [cited 2021 Apr 28]. Available from: https://www.gtexportal.org/home/

10. Consortium TGte. The GTEx Consortium atlas of genetic regulatory effects across human tissues. Science. 2020 Sep 11;369(6509):1318–30.

11. Das S, Forer L, Schönherr S, Sidore C, Locke AE, Kwong A, et al. Next-generation genotype imputation service and methods. Nat Genet. 2016 Oct;48(10):1284–7.

12. GTEx Consortium, Laboratory, Data Analysis &Coordinating Center (LDACC)—Analysis Working Group, Statistical Methods groups—Analysis Working Group, Enhancing GTEx (eGTEx) groups, NIH Common Fund, NIH/NCI, et al. Genetic effects on gene expression across human tissues. Nature. 2017 Oct 11;550(7675):204–13.

13. Robinson MD, Oshlack A. A scaling normalization method for differential expression analysis of RNA-seq data. Genome Biology. 2010 Mar 2;11(3):R25.

14. Poplin R, Ruano-Rubio V, DePristo MA, Fennell TJ, Carneiro MO, Auwera GAV der, et al. Scaling accurate genetic variant discovery to tens of thousands of samples. bioRxiv. 2018 Jul 24;201178.

15. Germline short variant discovery (SNPs + Indels) [Internet]. GATK. [cited 2021 May 20].

Available from: https://gatk.broadinstitute.org/hc/en-us/articles/360035535932-Germline-short-variant-discovery-SNPs-Indels-

16. Morris AP. Transethnic meta-analysis of genomewide association studies. Genet Epidemiol. 2011 Dec;35(8):809–22.

17. Li YR, Keating BJ. Trans-ethnic genome-wide association studies: advantages and challenges of mapping in diverse populations. Genome Med [Internet]. 2014 Oct 31 [cited 2021 Jun 7];6. Available from: https://www.ncbi.nlm.nih.gov/pmc/articles/PMC4254423/
