# Supplementary material for: Sequencing-based fine-mapping and in silico functional characterization of the 10q24.32 arsenic metabolism efficiency locus across multiple arsenic-exposed populations
Source: PLoS Genet. 2023 Jan 20;19(1):e1010588. doi: 10.1371/journal.pgen.1010588 (PMC9891528; doi:10.1371/journal.pgen.1010588)
Supplement: S1 Fig — a. Measures of total water arsenic (μg/L) measured for individuals in three arsenic-exposed populations. The Health Effect of Arsenic Longitudinal Study (HEALS, in red), the New Hampshire Skin Cancer Study (NHSCS, in blue). Measurements in HEALS are based off arsenic levels in wells frequented by the individual participants; in NHSCS measurements were taken from participants’ homes. (PDF) [file pgen.1010588.s002.pdf]

Fig S1 Arsenic Exposure Across Arsenic-Exposed Populations

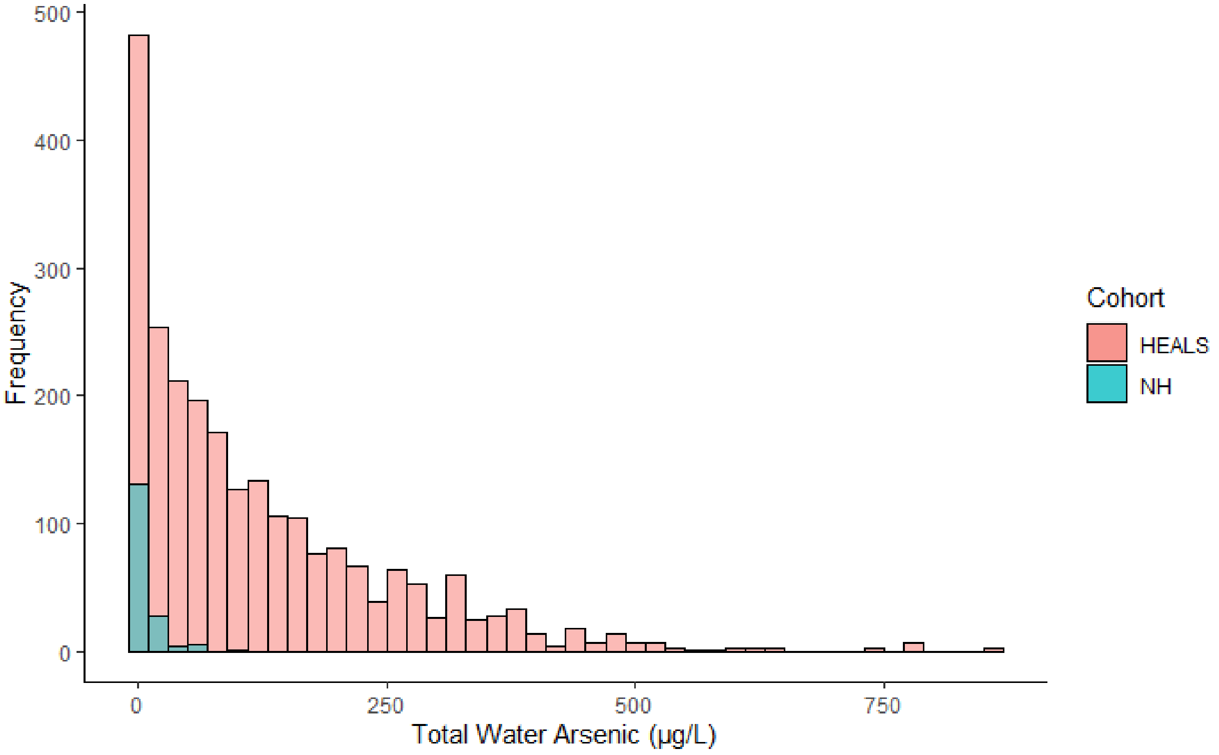

Fig S1. Arsenic Exposure Across Arsenic-Exposed Populations
Measures of total water arsenic (µg/L) measured for individuals in three arsenic-exposed populations. The Health Effect of Arsenic Longitudinal Study (HEALS, in red), the New Hampshire Skin Cancer Study (NHSCS, in blue). Measurements in HEALS are based off arsenic levels in wells frequented by the individual participants; in NHSCS measurements were taken from participants' homes.
