# Supplementary material for: Sequencing-based fine-mapping and in silico functional characterization of the 10q24.32 arsenic metabolism efficiency locus across multiple arsenic-exposed populations
Source: PLoS Genet. 2023 Jan 20;19(1):e1010588. doi: 10.1371/journal.pgen.1010588 (PMC9891528; doi:10.1371/journal.pgen.1010588)
Supplement: S2 Fig — a. DMA% distribution in three arsenic-exposed populations. The Health Effect of Arsenic Longitudinal Study (HEALS, in red), the New Hampshire Skin Cancer Study (NHSCS, in green), and the Strong Heart Study (SHS, in blue). (PDF) [file pgen.1010588.s003.pdf]

Fig S2 Distribution of urinary DMA percent across cohorts

A. HEALS DMA% Distribution

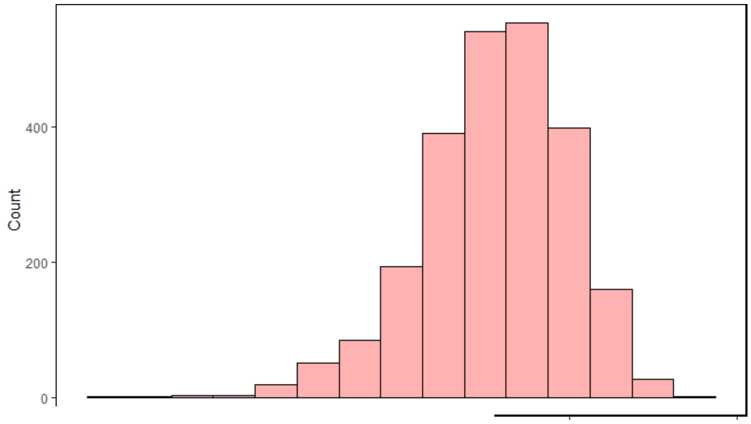

B. NHSCS DMA% Distribution

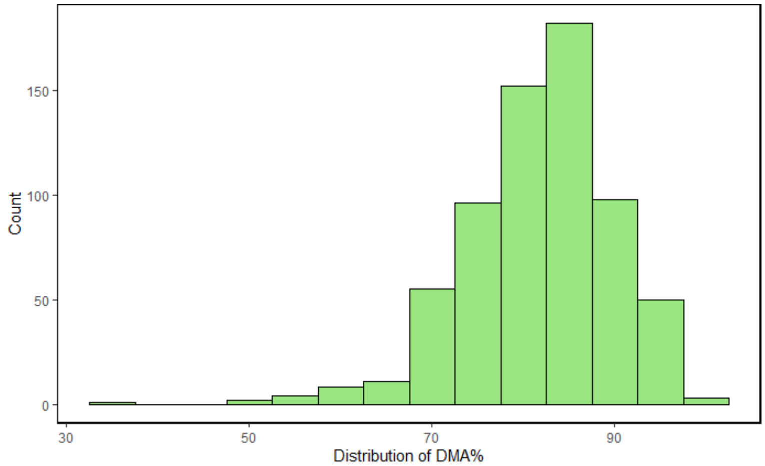

C. SHS DMA% Distribution

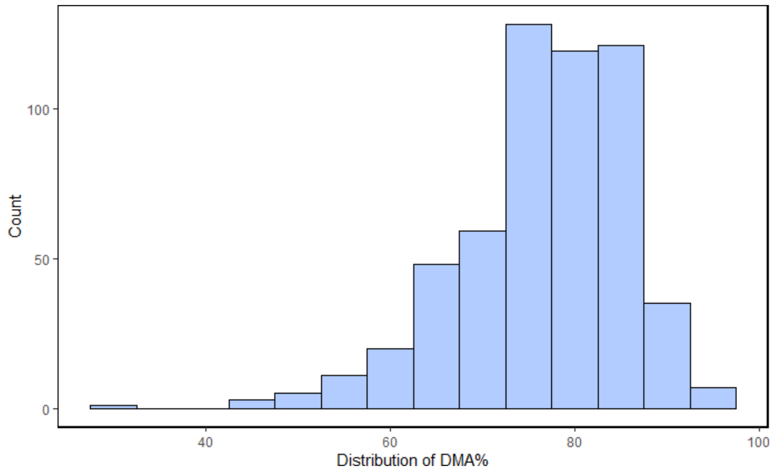

Fig S2. Distribution of urinary DMA percent across cohorts
DMA% distribution in three arsenic-exposed populations. The Health Effect of Arsenic Longitudinal Study (HEALS, in red), the New Hampshire Skin Cancer Study (NHSCS, in green), and the Strong Heart Study (SHS, in blue).
