# Supplementary material for: Sequencing-based fine-mapping and in silico functional characterization of the 10q24.32 arsenic metabolism efficiency locus across multiple arsenic-exposed populations
Source: PLoS Genet. 2023 Jan 20;19(1):e1010588. doi: 10.1371/journal.pgen.1010588 (PMC9891528; doi:10.1371/journal.pgen.1010588)
Supplement: S3 Fig — a. Results of a pre-imputation genetic association study of arsenic metabolism efficiency (DMA%) in the 10q24.32 region in three arsenic-exposed populations. P-values were generated with linear models adjusted for age and sex as well as kinship (HEALS) and population structure (SHS). The SNP with the strongest association is labeled in each panel. The top panel for each population shows the overall association results, the next shows p-values from models adjusted for the initial lead SNP, and the bottom panel shows the result of models adjusted for both previously identified variants. Three variants were identified in the Health Effect of Arsenic Longitudinal Study (HEALS), four in the Strong Heart Study (SHS), and one in New Hampshire Skin Cancer Study (NHSCS). (PDF) [file pgen.1010588.s004.pdf]

Fig S3 Non-Imputed DMA% Conditional Association Results

C. NH Conditional Association Results

B. SHS Conditional Association Results

A. HEALS Conditional Association Results

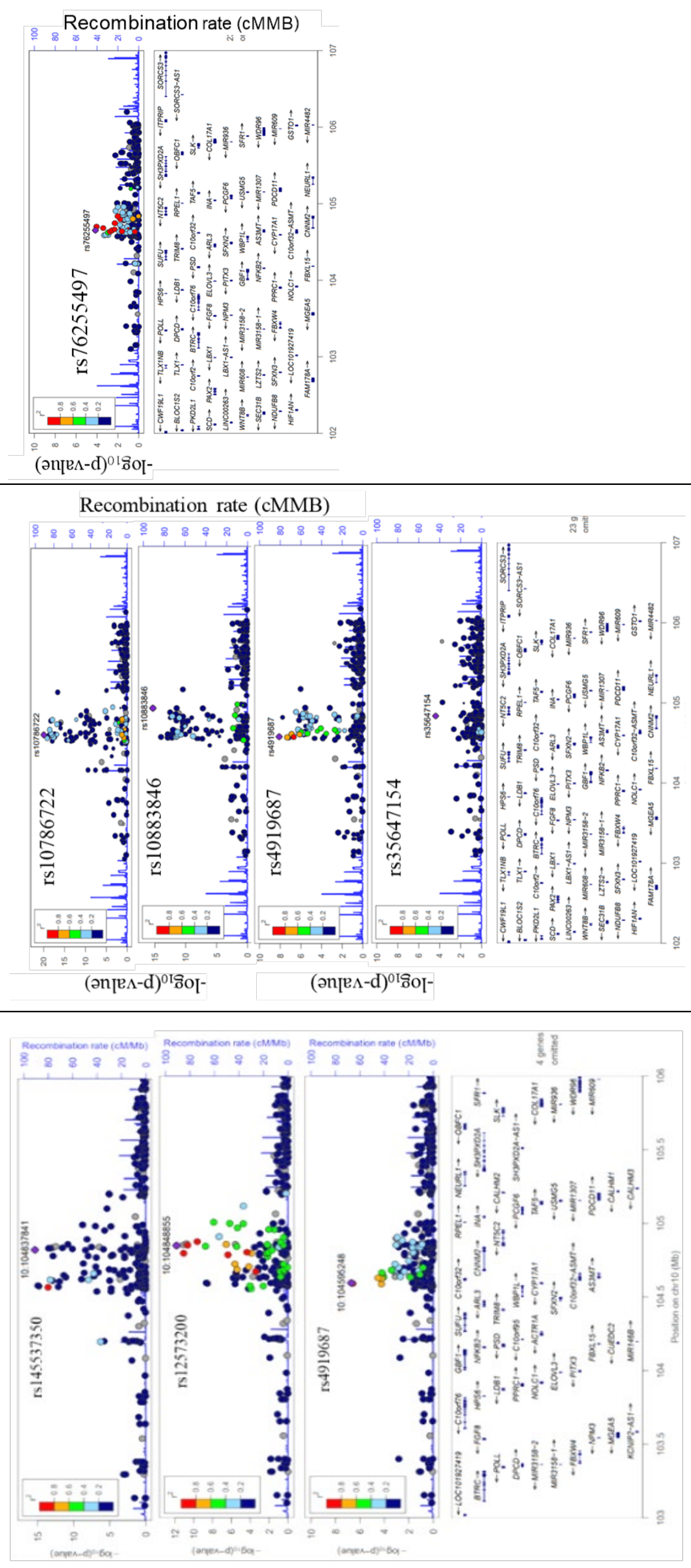

Fig S3. Non-imputed DMA% Conditional Association Results.
Results of a pre-imputation genetic association study of arsenic metabolism efficiency (DMA%) in the 10q24.32 region in three arsenic-exposed populations. P-values were generated with linear models adjusted for age and sex as well as kinship (HEALS) and population structure (SHS). The SNP with the strongest association is labeled in each panel. The top panel for each population shows the overall association results, the next shows p-values from models adjusted for the initial lead SNP, and the bottom panel shows the result of models adjusted for both previously identified variants. Three variants were identified in the Health Effect of Arsenic Longitudinal Study (HEALS), four in the Strong Heart Study (SHS), and one in New Hampshire Skin Cancer Study (NHSCS).
