# Supplementary material for: Sequencing-based fine-mapping and in silico functional characterization of the 10q24.32 arsenic metabolism efficiency locus across multiple arsenic-exposed populations
Source: PLoS Genet. 2023 Jan 20;19(1):e1010588. doi: 10.1371/journal.pgen.1010588 (PMC9891528; doi:10.1371/journal.pgen.1010588)
Supplement: S4 Fig — a. Results of a post-imputation genetic association study of MMA% in the 10q24.32 region in HEALS. P-values were generated with linear models adjusted for age, sex, and kinship. The top panel represents overall association results, the second shows p-values from models adjusted for the initial lead SNP, and the bottom panel shows the result of models adjusted for both previously identified variants. Two lead variants were identified in this analysis: chr10:102842818 and chr10:102888092. We further note that the primary signal likely captures the tertiary signal identified in the analysis of DMA%. (PDF) [file pgen.1010588.s005.pdf]

**Fig S4.** HEALS MMA% Conditional Association Results

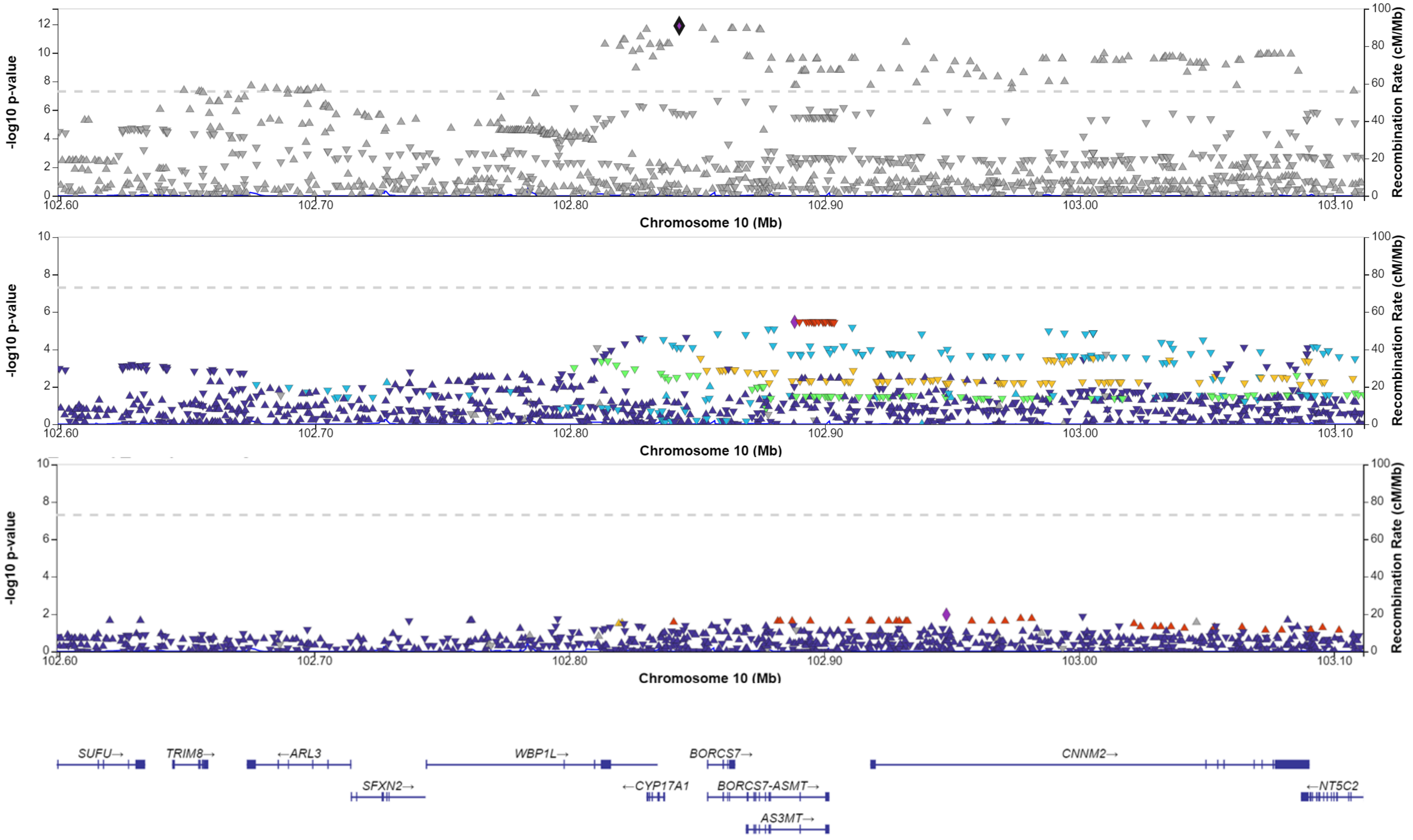

**Fig S4.** HEALS MMA% Conditional Association Results

Results of a post-imputation genetic association study of MMA% in the 10q24.32 region in HEALS. P-values were generated with linear models adjusted for age, sex, and kinship. The top panel represents overall association results, the second shows p-values from models adjusted for the initial lead SNP, and the bottom panel shows the result of models adjusted for both previously identified variants. Two lead variants were identified in this analysis: chr10:102842818 and chr10:102888092. We further note that the primary signal likely captures the tertiary signal identified in the analysis of DMA%.
