# Supplementary material for: Sequencing-based fine-mapping and in silico functional characterization of the 10q24.32 arsenic metabolism efficiency locus across multiple arsenic-exposed populations
Source: PLoS Genet. 2023 Jan 20;19(1):e1010588. doi: 10.1371/journal.pgen.1010588 (PMC9891528; doi:10.1371/journal.pgen.1010588)
Supplement: S6 Fig — a. Results of a post-imputation genetic association study of MMA% in the 10q24.32 region in SHS. P-values were generated with linear models adjusted for age, sex, and population structure. The top panel represents overall association results, the second shows p-values from models adjusted for the initial lead SNP, and the bottom panel shows the result of models adjusted for both previously identified variants. Two lead variants were identified in this analysis: chr10:103078084 (rs145537350) and chr10:102842863 (rs4919688). We further note that the second signal is identical to the secondary signal identified in the analysis of DMA% and that the lead DMA% signal was the second most significant signal in the primary analysis and is in strong LD with the identified lead variant. (PDF) [file pgen.1010588.s007.pdf]

Fig S6. SHS MMA% Conditional Association Results

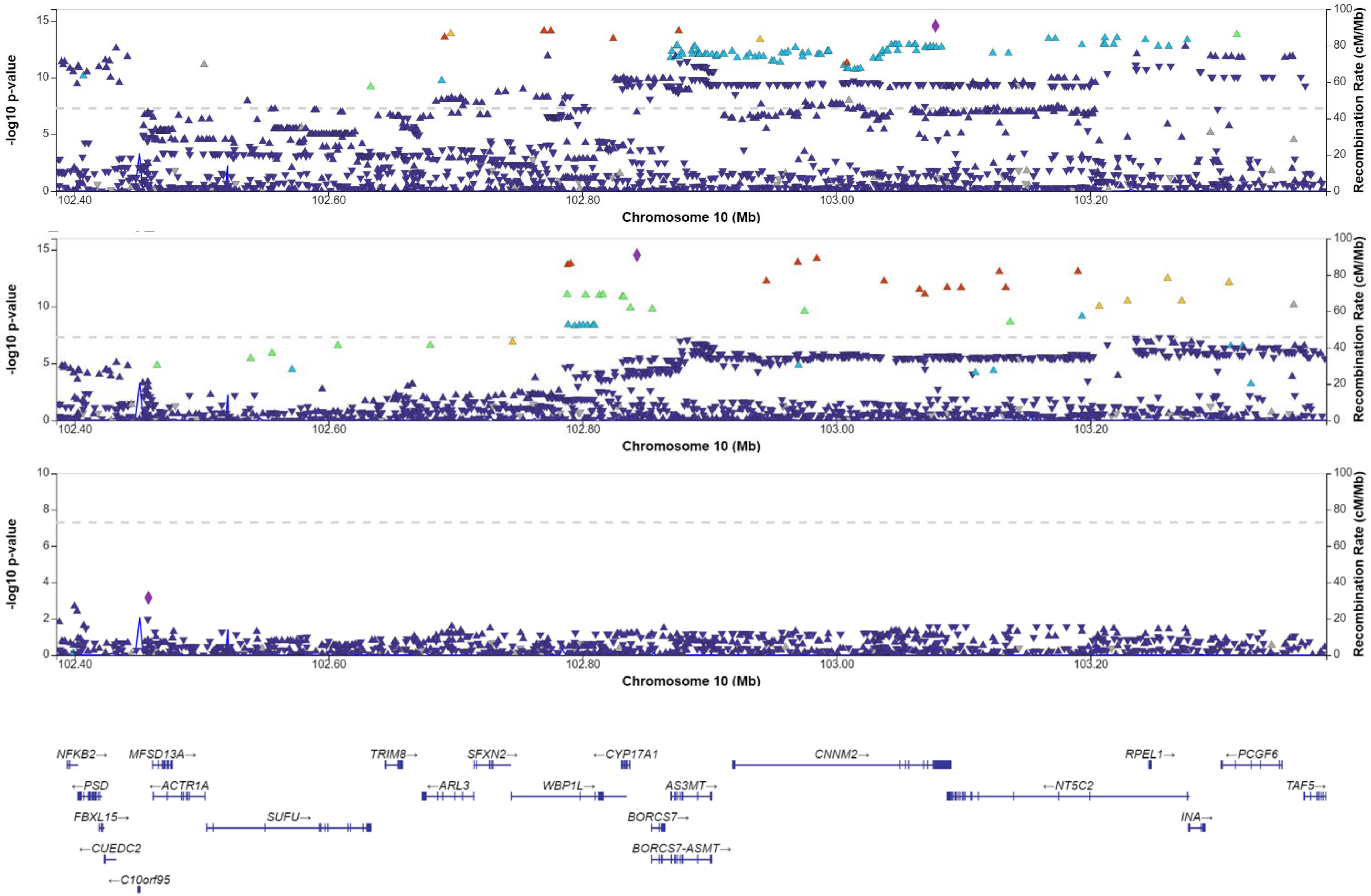

Fig S6. SHS MMA% Conditional Association Results

Results of a post-imputation genetic association study of MMA% in the 10q24.32 region in SHS. P-values were generated with linear models adjusted for age, sex, and population structure. The top panel represents overall association results, the second shows p-values from models adjusted for the initial lead SNP, and the bottom panel shows the result of models adjusted for both previously identified variants. Two lead variants were identified in this analysis: chr10:103078084 (rs145537350) and chr10:102842863 (rs4919688). We further note that the second signal is identical to the secondary signal identified in the analysis of DMA% and that the lead DMA% signal was the second most significant signal in the primary analysis and is in strong LD with the identified lead variant.
