# Supplementary material for: Sequencing-based fine-mapping and in silico functional characterization of the 10q24.32 arsenic metabolism efficiency locus across multiple arsenic-exposed populations
Source: PLoS Genet. 2023 Jan 20;19(1):e1010588. doi: 10.1371/journal.pgen.1010588 (PMC9891528; doi:10.1371/journal.pgen.1010588)
Supplement: S8 Fig — a. In both cohorts, we observe a negative correlation in which higher exposure is associated with lower DMA%. (PDF) [file pgen.1010588.s009.pdf]

Fig S8 Correlation of DMA% with arsenic exposure (based on drinking water arsenic concentration) **A. Correlation in HEALS**

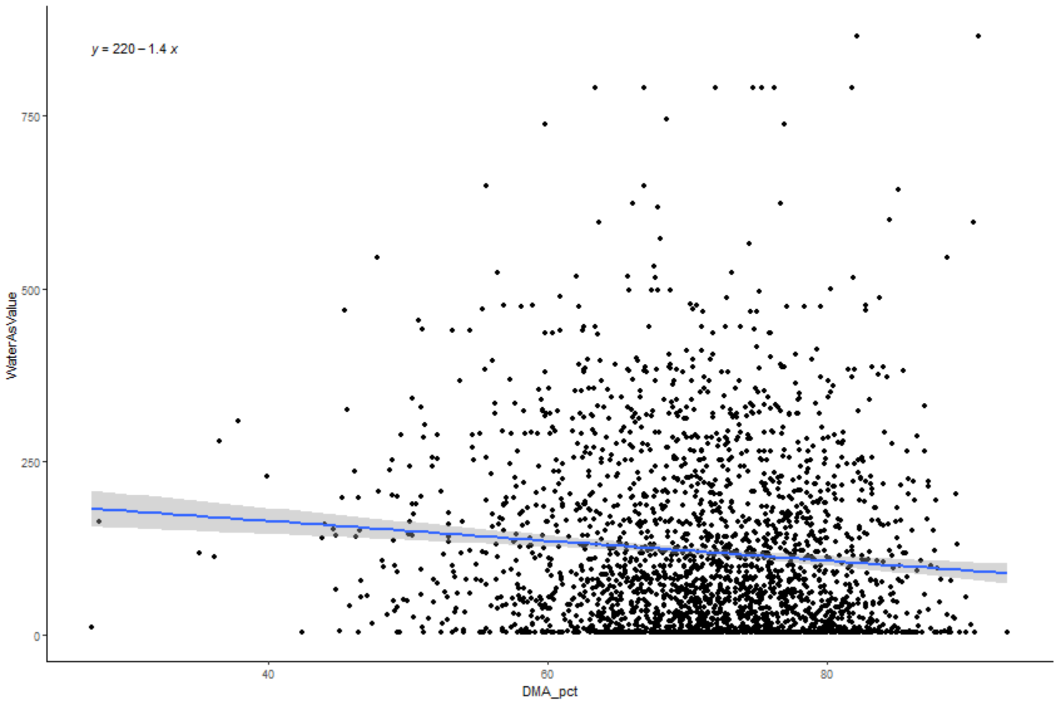

B. Correlation in NHSCS

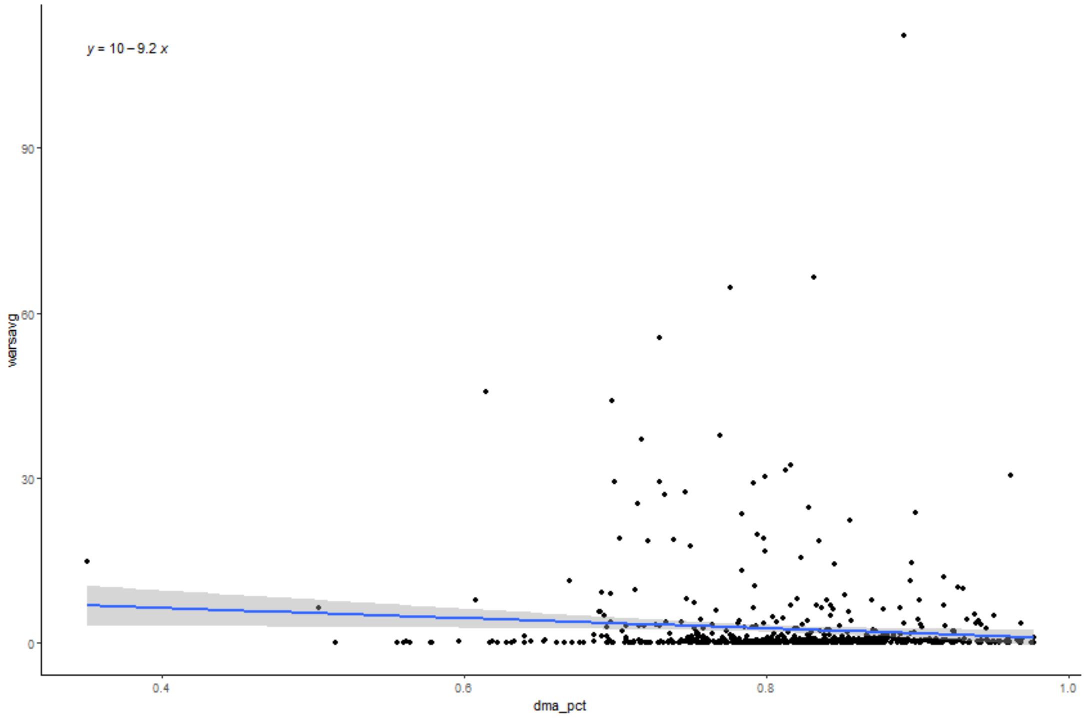

Fig S8. Correlation of DMA% with arsenic exposure (based on drinking water arsenic concentration) in HEALS **(A)** and NHSCS **(B)**. In both cohorts, we observe a negative correlation in which higher exposure is associated with lower DMA%.
