# Supplementary material for: Sequencing-based fine-mapping and in silico functional characterization of the 10q24.32 arsenic metabolism efficiency locus across multiple arsenic-exposed populations
Source: PLoS Genet. 2023 Jan 20;19(1):e1010588. doi: 10.1371/journal.pgen.1010588 (PMC9891528; doi:10.1371/journal.pgen.1010588)
Supplement: S9 Fig — a. Results of in silico-functional analysis reveals overlap with genomic annotations for candidate causal SNPs from HEALS confidence set 1, corresponding to the primary association signal (lead SNP rs145537350). Multiple SNPs in the confidence set overlap genomic features including candidate cis-regulatory elements and transcription factor binding sites. Highlighted panels show details of candidate causal SNPs overlapping these features. (PDF) [file pgen.1010588.s010.pdf]

Fig S9

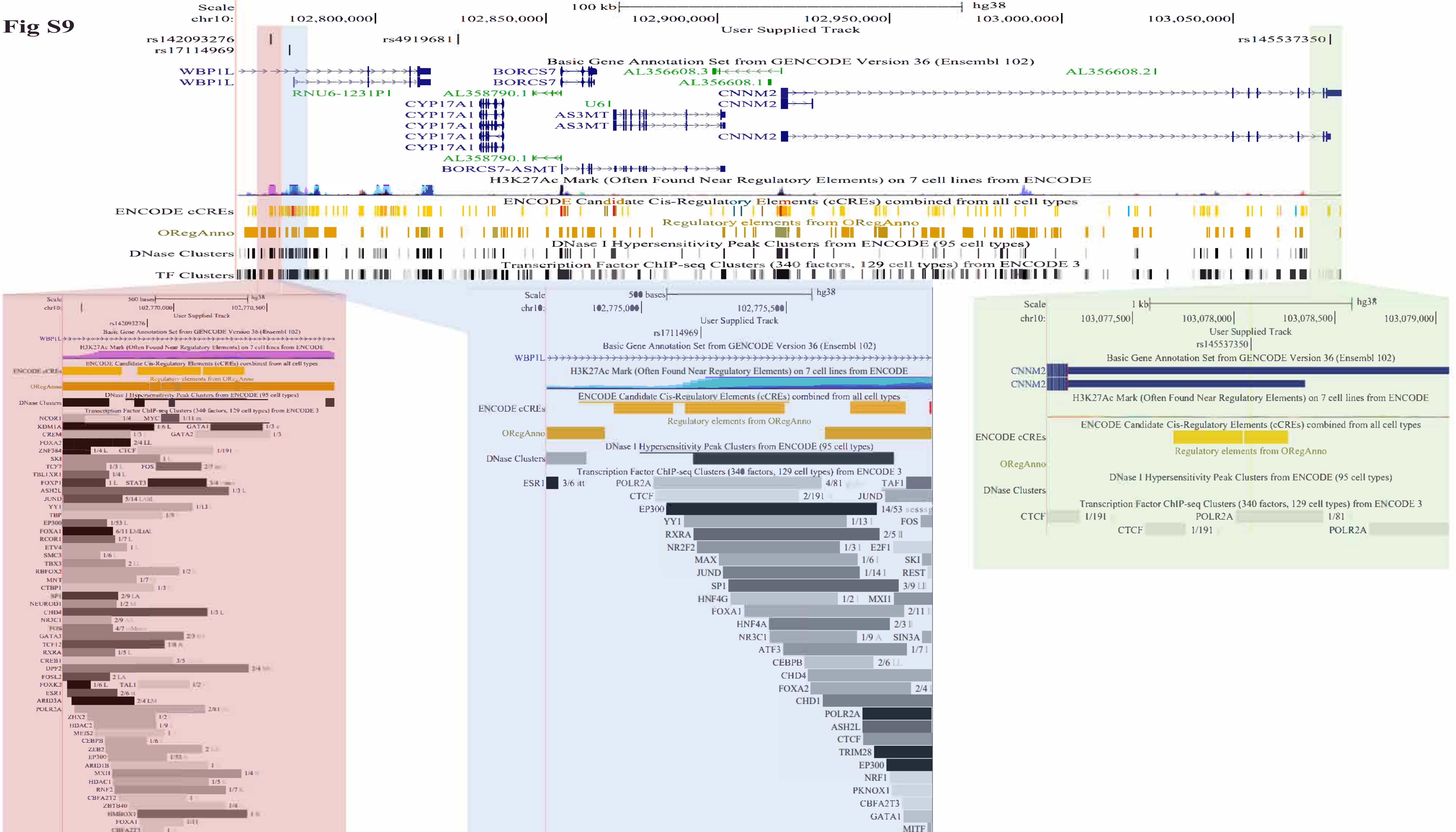

Scale
chr10:
100 kb
hg38
102,800,000
102,850,000
102,900,000
102,950,000
103,000,000
103,050,000
User Supplied Track
rs142093276
rs17114969
rs4919681
rs145537350
Basic Gene Annotation Set from GENCODE Version 36 (Ensembl 102)
WBP1L
BORCS7
AL356608.3
AL356608.2
AL356608.1
RNU6-1231P
AL358790.1
CNNM2
CYP17A1
U6
AS3MT
BORCS7-ASMT
H3K27Ac Mark (Often Found Near Regulatory Elements) on 7 cell lines from ENCODE
ENCODE Candidate Cis-Regulatory Elements (cCREs) combined from all cell types
ENCODE cCREs
Regulatory elements from ORegAnno
ORegAnno
DNase I Hypersensitivity Peak Clusters from ENCODE (95 cell types)
DNase Clusters
Transcription Factor ChIP-seq Clusters (340 factors, 129 cell types) from ENCODE 3
TF Clusters
500 bases
102,775,000
102,775,500
ESR1
POLR2A
TAF1
CTCF
JUND
EP300
YY1
FOS
RXRA
NR2F2
E2F1
MAX
SKI
REST
SP1
HNF4G
MXI1
FOXA1
HNF4A
NR3C1
SIN3A
ATF3
CEBPB
CHD4
FOXA2
CHD1
ASH2L
TRIM28
NRF1
PKNOX1
CBFA2T3
GATA1
MITF
1 kb
103,077,500
103,078,000
103,078,500
103,079,000

**Fig S9.** Genomic annotations of HEALS confidence set 1.

Results of in silico-functional analysis reveals overlap with genomic annotations for candidate causal SNPs from HEALS confidence set 1, corresponding to the primary association signal (lead SNP rs145537350). Multiple SNPs in the confidence set overlap genomic features including candidate cis-regulatory elements and transcription factor binding sites. Highlighted panels show details of candidate causal SNPs overlapping these features.
