# Supplementary material for: Sequencing-based fine-mapping and in silico functional characterization of the 10q24.32 arsenic metabolism efficiency locus across multiple arsenic-exposed populations
Source: PLoS Genet. 2023 Jan 20;19(1):e1010588. doi: 10.1371/journal.pgen.1010588 (PMC9891528; doi:10.1371/journal.pgen.1010588)
Supplement: S10 Fig — a. A. The distribution of AS3MT expression across human tissues reveals higher expression in the adrenal gland compared with all other available tissues. B. With the removal of the adrenal gland, we can see some variation in AS3MT expression across tissue types. Fig produced using the GTEx portal. (PDF) [file pgen.1010588.s011.pdf]

Fig S10 Distribution of *AS3MT* expression across human tissues

A. AS3MT expression across tissues

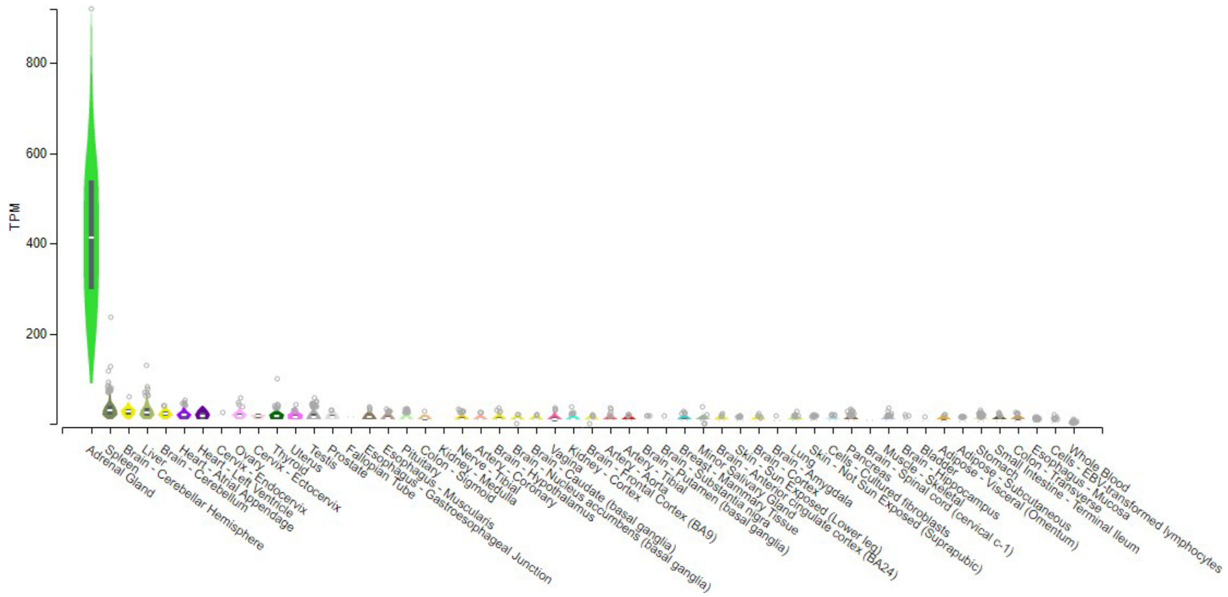

B. AS3MT expression across tissues excluding the adrenal gland

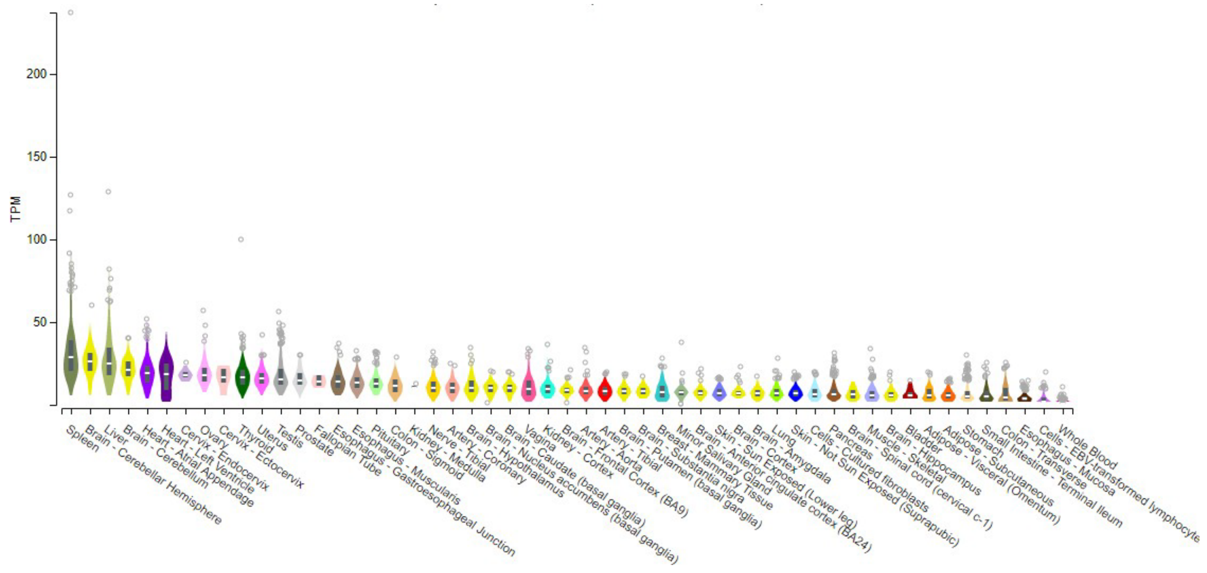

Fig S10. Distribution of AS3MT expression across human tissues.
A. The distribution of AS3MT expression across human tissues reveals higher expression in the adrenal gland compared with all other available tissues. **B.** With the removal of the adrenal gland, we can see some variation in AS3MT expression across tissue types. Fig produced using the GTEx portal
