# Supplementary material for: Sequencing-based fine-mapping and in silico functional characterization of the 10q24.32 arsenic metabolism efficiency locus across multiple arsenic-exposed populations
Source: PLoS Genet. 2023 Jan 20;19(1):e1010588. doi: 10.1371/journal.pgen.1010588 (PMC9891528; doi:10.1371/journal.pgen.1010588)
Supplement: S11 Fig — a. We detect AS3MT eQTLs in the liver and observe evidence of co-localization (Panel A), but this evidence was not consistent across all sets of priors analyzed. The low efficiency (low DMA%) allele at rs4919687 is associated with lower AS3MT expression in the liver, though this pattern is observed more strongly in other tissue types (Panel B). (PDF) [file pgen.1010588.s012.pdf]

Fig S11 Co-localization between HEALS DMA% association signal (rs4919687) and AS3MT eQTLs in the liver

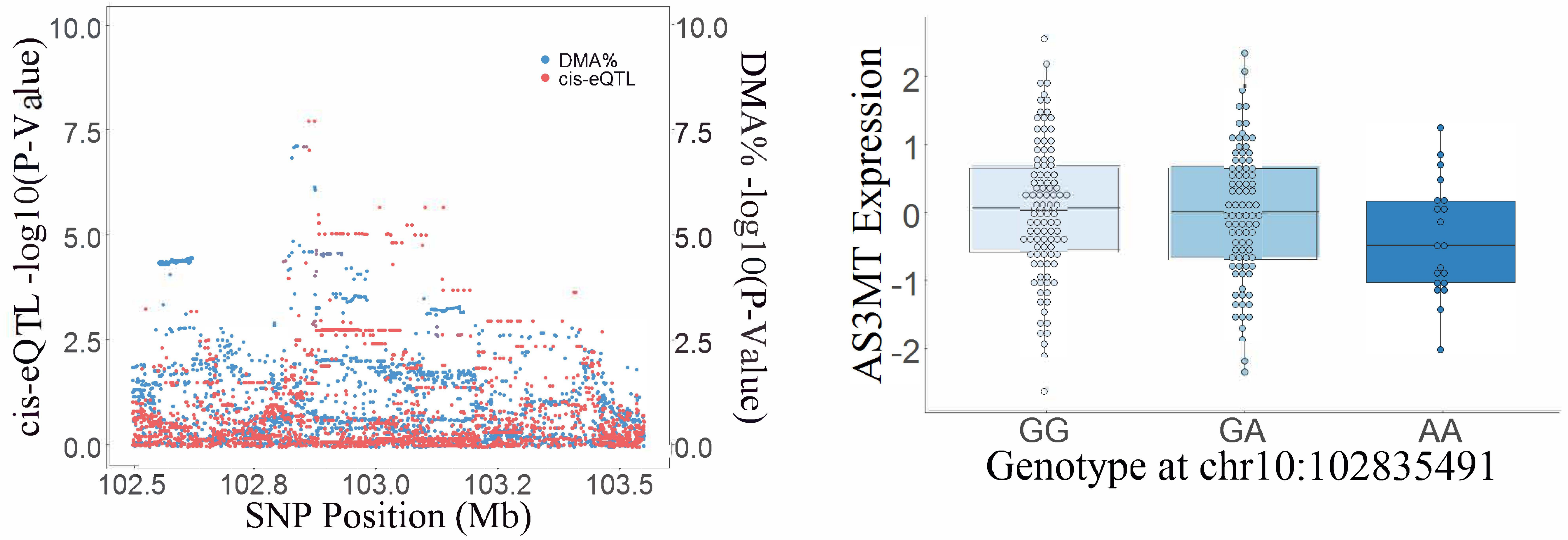

Fig S11. Co-localization between HEALS DMA% association signal (rs4919687) and *AS3MT* eQTLs in the Liver

We detect *AS3MT* eQTLs in the liver and observe evidence of co-localization (Panel A), but this evidence was not consistent across all sets of priors analyzed. The low efficiency (low DMA%) allele at rs4919687 is associated with lower *AS3MT* expression in the liver, though this pattern is observed more strongly in other tissue types (Panel B).
