# Supplementary material for: Sequencing-based fine-mapping and in silico functional characterization of the 10q24.32 arsenic metabolism efficiency locus across multiple arsenic-exposed populations
Source: PLoS Genet. 2023 Jan 20;19(1):e1010588. doi: 10.1371/journal.pgen.1010588 (PMC9891528; doi:10.1371/journal.pgen.1010588)
Supplement: S1 Table — (DOCX) [file pgen.1010588.s013.docx]

**Table S1** Minor Allele Frequency (MAF) comparison of lead signals across SHS centers

| **Lead SNP** | **SHS DK Center MAF** | **SHS OK Center MAF** | **SHS AZ Center MAF** |
| --- | --- | --- | --- |
| rs145537350 | 0.159 | 0.121 | 0.151 |
| rs12573221 | 0.004 | 0 | 0 |
| rs4919687 | 0.236 | 0.212 | 0.187 |
| rs191177668 | 0.157 | 0.123 | 0.157 |
| rs4919688 | 0.252 | 0.221 | 0.482 |

Abbreviations: SNP, single nucleotide polymorphism; MAF, minor allele frequency; HEALS, Health Effects of Arsenic Longitudinal Study; SHS, Strong Heart Study; NH, Hew Hampshire Case-Control Study of Squamous Cell Carcinoma; AZ, Arizona; OK, Oklahoma; DK, Dakota. *Indicates SNPs that are in high LD with each other across multiple reference population including Bengali, Ad-Mixed Americans, and Europeans. ** Refers to the association-based SHS confidence set
