# Supplementary material for: Sequencing-based fine-mapping and in silico functional characterization of the 10q24.32 arsenic metabolism efficiency locus across multiple arsenic-exposed populations
Source: PLoS Genet. 2023 Jan 20;19(1):e1010588. doi: 10.1371/journal.pgen.1010588 (PMC9891528; doi:10.1371/journal.pgen.1010588)
Supplement: S2 Table — (DOCX) [file pgen.1010588.s014.docx]

**Table S2** HEALS Confidence Sets (C.S.) from population-specific fine- mapping analysis

**S2A.** HEALS Confidence Set corresponding to primary HEALS association signal (HEALS Confidence Set 1)

| Variant | rsID | MAF | Posterior Inclusion Probability |
| --- | --- | --- | --- |
| chr10:103078084:G:C* | rs145537350 | 0.007 | 0.836 |
| chr10:102769853:T:C | rs17114969 | 0.008 | 0.052 |
| chr10:102775204:G:A | rs17114969 | 0.008 | 0.052 |
| chr10:102824339:C:T* | rs4919681 | 0.007 | 0.052 |

*Indicates SNPs seen in SHS Confidence Set

**S2B.** HEALS Confidence Set corresponding to tertiary HEALS association signal (HEALS Confidence Set 2)

| Variant | rsID | MAF | Posterior Inclusion Probability |
| --- | --- | --- | --- |
| chr10:102835149:T:G* | rs743575 | 0.123 | 0.189 |
| chr10:102838165:C:T* | rs10883784 | 0.121 | 0.16 |
| chr10:102838849:G:C* | rs10786714 | 0.121 | 0.16 |
| chr10:102831395:T:A* | rs10883783 | 0.122 | 0.154 |
| chr10:102835491:G:A* | rs4919687 | 0.122 | 0.143 |
| chr10:102856743:T:C | rs4919690 | 0.115 | 0.087 |
| chr10:102827267:A:G* | rs4919684 | 0.121 | 0.083 |

*Indicates SNPs seen in SHS Confidence Set

**S2C.** HEALS Confidence Set corresponding to secondary HEALS association signal (HEALS Confidence Set 3)

| Variant | rsID | MAF | Posterior Inclusion Probability |  |
| --- | --- | --- | --- | --- |
| chr10:103200543:G:A | rs144453732 | 0.125 | 0.083 |  |
| chr10:103201099:C:G | rs75075426 | 0.125 | 0.083 |  |
| chr10:103197395:T:C | rs1317560071 | 0.124 | 0.077 |  |
| chr10:103179295:C:T | rs114373406 | 0.125 | 0.067 |  |
| chr10:103182486:G:A | rs184211672 | 0.125 | 0.067 |  |
| chr10:103089387:A:C | rs12573221 | 0.134 | 0.061 |  |
| chr10:103101706:AT:A |  | 0.131 | 0.048 |  |
| chr10:103093001:AG:A | rs2067306716 | 0.134 | 0.035 |  |
| chr10:103107938:T:G | rs140814859 | 0.131 | 0.03 |  |
| chr10:103115547:A:C | rs7908960 | 0.131 | 0.03 |  |
| chr10:103120099:A:G | rs2148199 | 0.130 | 0.03 |  |
| chr10:103089098:A:T | rs12573200 | 0.133 | 0.029 |  |
| chr10:103132459:T:C | rs75287094 | 0.130 | 0.027 |  |
| chr10:103089087:A:T | rs12573199 | 0.132 | 0.025 |  |
| chr10:102947977:C:T | rs149177817 | 0.128 | 0.025 |  |
| chr10:103091643:G:A | rs79438485 | 0.136 | 0.021 |  |
| chr10:102879630:G:A | rs187598077 | 0.129 | 0.019 |  |
| chr10:103036487:G:A | | rs78312046 | 0.136 | 0.018 |
| chr10:102907267:AG:A | |  | 0.128 | 0.016 |
| chr10:103140364:A:AT | | rs35653865 | 0.129 | 0.016 |
| chr10:103141084:C:T | | rs78436955 | 0.128 | 0.016 |
| chr10:103165786:C:G | | rs146240286 | 0.128 | 0.014 |
| chr10:103168407:G:C | | rs75839032 | 0.128 | 0.014 |
| chr10:103164282:G:A | | rs149555487 | 0.127 | 0.01 |
| chr10:103225318:CAG:C | |  | 0.061 | 0.007 |
| chr10:102868867:A:G | | rs17115179 | 0.129 | 0.007 |
| chr10:102857890:C:T | | rs76429823 | 0.130 | 0.007 |
| chr10:103240962:G:A | | rs140708661 | 0.061 | 0.006 |
| chr10:102894115:G:A | | rs78561456 | 0.124 | 0.004 |
| chr10:103097448:GA:G | | rs747089327 | 0.160 | 0.004 |
| chr10:103097461:A:C | | rs10786737 | 0.161 | 0.004 |
| chr10:103051328:G:C | | rs141482908 | 0.127 | 0.004 |
| chr10:103043557:T:C | | rs17115381 | 0.127 | 0.003 |
| chr10:103242977:G:T | | rs141435397 | 0.062 | 0.003 |
| chr10:102845572:G:A | | rs79424856 | 0.130 | 0.003 |
| chr10:103004391:A:G | | rs77426338 | 0.128 | 0.003 |
| chr10:103002173:A:G | | rs12779991 | 0.165 | 0.002 |
| chr10:103005180:A:C | | rs369395328 | 0.165 | 0.002 |
| chr10:103005181:T:A | | rs372721431 | 0.165 | 0.002 |
| chr10:103005182:A:G | | rs377385988 | 0.165 | 0.002 |
| chr10:103005192:A:G | | rs11191500 | 0.165 | 0.002 |
| chr10:103005194:C:G | | rs11191501 | 0.165 | 0.002 |
| chr10:102900964:A:G | | rs17881031 | 0.124 | 0.002 |
| chr10:102903657:T:C | | rs140973522 | 0.123 | 0.002 |
| chr10:102877791:C:T | | rs11191435 | 0.162 | 0.002 |
| chr10:102879981:G:A | | rs12774047 | 0.162 | 0.002 |
| chr10:103010229:C:CT | |  | 0.128 | 0.002 |
| chr10:103048302:G:T | | rs12416483 | 0.164 | 0.002 |
| chr10:103238997:A:T | | rs114710011 | 0.065 | 0.002 |
| chr10:102957002:A:T | | rs116050393 | 0.125 | 0.002 |
