# Supplementary material for: Sequencing-based fine-mapping and in silico functional characterization of the 10q24.32 arsenic metabolism efficiency locus across multiple arsenic-exposed populations
Source: PLoS Genet. 2023 Jan 20;19(1):e1010588. doi: 10.1371/journal.pgen.1010588 (PMC9891528; doi:10.1371/journal.pgen.1010588)
Supplement: S3 Table — (DOCX) [file pgen.1010588.s015.docx]

**Table S3** SHS Confidence Sets (C.S.) from population-specific fine- mapping analysis based on summary association statistics

**S3A.** SHS Confidence Set corresponding to primary SHS association signal (SHS Association-Based Confidence Set 1)

| Variant | rsID | MAF | Posterior Inclusion Probability |
| --- | --- | --- | --- |
| chr10:102825815:G:A | rs35756290 | 0.211 | 0.005 |
| chr10:102827267:A:G* | rs4919684 | 0.217 | 0.0172 |
| chr10:102827605:G:T | rs4919685 | 0.219 | 0.01 |
| chr10:102829990:C:T | rs12254149 | 0.218 | 0.01 |
| chr10:102831395:T:A* | rs10883783 | 0.220 | 0.01 |
| chr10:102835149:T:G* | rs743575 | 0.219 | 0.01 |
| chr10:102835491:G:A* | rs4919687 | 0.220 | 0.01 |
| chr10:102838165:C:T* | rs10883784 | 0.218 | 0.01 |
| chr10:102838849:G:C* | rs10786714 | 0.218 | 0.01 |
| chr10:102842818:G:T | rs11191415 | 0.219 | 0.005 |
| chr10:102870424:T:C | rs4917986 | 0.173 | 0.004 |
| chr10:102874350:A:G | rs10509760 | 0.172 | 0.021 |
| chr10:102874717:A:G | rs3740394 | 0.172 | 0.021 |
| chr10:102875930:A:T | rs191177668 | 0.143 | 0.140 |
| chr10:102878966:T:C | rs11191439 | 0.172 | 0.004 |
| chr10:102886187:C:A | rs12245779 | 0.172 | 0.0124 |
| chr10:102886540:T:C | rs12253834 | 0.172 | 0.004 |
| chr10:102888017:A:T | rs77505796 | 0.167 | 0.006 |
| chr10:102888709:C:T | rs112507051 | 0.167 | 0.006 |
| chr10:102890873:G:A | rs17882560 | 0.172 | 0.015 |
| chr10:102891298:C:T | rs11191445 | 0.172 | 0.005 |
| chr10:102891723:A:G | rs11191446 | 0.173 | 0.005 |
| chr10:102892198:T:C | rs76255497 | 0.173 | 0.005 |
| chr10:102892288:C:T | rs75691516 | 0.174 | 0.005 |
| chr10:102896558:C:T | rs113320965 | 0.173 | 0.005 |
| chr10:102896914:T:C | rs80327774 | 0.173 | 0.005 |
| chr10:102898415:G:A | rs111638521 | 0.173 | 0.005 |
| chr10:102902240:C:T | rs7084472 | 0.173 | 0.005 |
| chr10:102905388:G:C | rs12253284 | 0.172 | 0.004 |
| chr10:102905640:T:C | rs12261040 | 0.173 | 0.003359 |
| chr10:102910589:C:T | rs112809537 | 0.172 | 0.005 |
| chr10:102911702:A:C | rs12251035 | 0.172 | 0.005 |
| chr10:102957014:A:T | rs113541728 | 0.170 | 0.005 |
| chr10:102961964:CTT:C | rs764945861 | 0.169 | 0.007 |
| chr10:102967767:G:T | rs12258949 | 0.168 | 0.033 |
| chr10:102985417:T:A | rs11191490 | 0.173 | 0.005 |
| chr10:102986711:C:T | rs113375453 | 0.172 | 0.005 |
| chr10:102993073:T:G | rs4917988 | 0.172 | 0.004 |
| chr10:102993074:A:T | rs4917379 | 0.172 | 0.004 |
| chr10:103043305:C:A | rs12257935 | 0.171 | 0.006 |
| chr10:103045853:C:A | rs35159404 | 0.171 | 0.006 |
| chr10:103047127:G:A | rs12266291 | 0.171 | 0.006 |
| chr10:103049240:G:A | rs12241091 | 0.171 | 0.006 |
| chr10:103057274:C:T | rs12246689 | 0.172 | 0.006 |
| chr10:103062252:C:T | rs77827514 | 0.172 | 0.006 |
| chr10:103070036:G:A | rs11191545 | 0.172 | 0.014 |
| chr10:103070877:C:T | rs12264456 | 0.171 | 0.019 |
| chr10:103073404:C:T | rs12257941 | 0.172 | 0.014 |
| chr10:103074165:A:G | rs61120766 | 0.172 | 0.014 |
| chr10:103076344:C:A | rs17727391 | 0.173 | 0.014 |
| chr10:103078084:G:C* | rs145537350 | 0.143 | 0.03 |
| chr10:103078753:G:A | rs4917382 | 0.173 | 0.014 |
| chr10:103082779:G:GTC | rs113304525 | 0.173 | 0.014 |
| chr10:103167119:G:A | rs112224955 | 0.160 | 0.015 |
| chr10:103172305:G:A | rs113973959 | 0.160 | 0.015 |
| chr10:103193264:C:A | rs111622998 | 0.165 | 0.008 |
| chr10:103210399:C:A | rs111668583 | 0.163 | 0.005 |
| chr10:103243262:G:T | rs78384860 | 0.158 | 0.081 |
| chr10:103253390:A:C | rs4918001 | 0.163 | 0.041 |
| chr10:103261717:C:T | rs7903472 | 0.163 | 0.041 |
| chr10:103274761:C:T | rs112069023 | 0.163 | 0.041 |
| chr10:103276197:A:G | rs80020194 | 0.158 | 0.081 |
| chr10:103315314:T:C | rs117848719 | 0.135 | 0.006 |

*Indicates SNPs identified in HEALS confidence sets.

**S3B.** SHS Confidence Set corresponding to secondary SHS association signal

| Variant | rsID | MAF | Posterior Inclusion Probability |
| --- | --- | --- | --- |
| chr10:102842863:C:T | rs4919688 | 0.268 | 0.316612 |
| chr10:102969519:C:T | rs4919696 | 0.272 | 0.165881 |
| chr10:102984468:G:A | rs17115317 | 0.273 | 0.100301 |
| chr10:103128280:C:T | rs546234534 | 0.272 | 0.185661 |
| chr10:103190143:G:C | rs145884725 | 0.274 | 0.185661 |
