# Supplementary material for: Sequencing-based fine-mapping and in silico functional characterization of the 10q24.32 arsenic metabolism efficiency locus across multiple arsenic-exposed populations
Source: PLoS Genet. 2023 Jan 20;19(1):e1010588. doi: 10.1371/journal.pgen.1010588 (PMC9891528; doi:10.1371/journal.pgen.1010588)
Supplement: S4 Table — (DOCX) [file pgen.1010588.s016.docx]

**Table S4** SHS Confidence Sets (C.S.) from population-specific fine- mapping analysis based on primary sequencing data

**S4A.** SHS Confidence Set corresponding to primary SHS association signal

| Variant | rsID | MAF | Posterior Inclusion Probability |
| --- | --- | --- | --- |
| chr10:102691571:A:G | rs148658224 | 0.142 | 0.003235 |
| chr10:102824339:C:T* | rs4919681 | 0.144 | 0.004987 |
| chr10:102870424:T:C | rs4917986 | 0.173 | 0.002311 |
| chr10:102873533:G:A | rs12259506 | 0.175 | 0.003484 |
| chr10:102874350:A:G | rs10509760 | 0.172 | 0.008703 |
| chr10:102874717:A:G | rs3740394 | 0.172 | 0.008703 |
| chr10:102875930:A:T | rs191177668 | 0.143 | 0.117945 |
| chr10:102876001:AG:A | rs747586333 | 0.175 | 0.003484 |
| chr10:102878966:T:C | rs11191439 | 0.172 | 0.003367 |
| chr10:102886187:C:A | rs12245779 | 0.172 | 0.005125 |
| chr10:102886540:T:C | rs12253834 | 0.172 | 0.003367 |
| chr10:102888017:A:T | rs77505796 | 0.167 | 0.007022 |
| chr10:102888709:C:T | rs112507051 | 0.167 | 0.007022 |
| chr10:102890873:G:A | rs17882560 | 0.172 | 0.004018 |
| chr10:102891298:C:T | rs11191445 | 0.172 | 0.002669 |
| chr10:102891723:A:G | rs11191446 | 0.173 | 0.002669 |
| chr10:102892198:T:C | rs76255497 | 0.173 | 0.002669 |
| chr10:102892288:C:T | rs75691516 | 0.174 | 0.002669 |
| chr10:102896558:C:T | rs113320965 | 0.173 | 0.002669 |
| chr10:102896914:T:C | rs80327774 | 0.173 | 0.002669 |
| chr10:102898415:G:A | rs111638521 | 0.173 | 0.002669 |
| chr10:102902240:C:T | rs7084472 | 0.173 | 0.002669 |
| chr10:102905388:G:C | rs12253284 | 0.172 | 0.00358 |
| chr10:102905640:T:C | rs12261040 | 0.173 | 0.002675 |
| chr10:102906131:C:T | rs112255065 | 0.172 | 0.002675 |
| chr10:102910589:C:T | rs112809537 | 0.172 | 0.002669 |
| chr10:102911702:A:C | rs12251035 | 0.172 | 0.002669 |
| chr10:102917905:G:C | rs74376228 | 0.166 | 0.007122 |
| chr10:102921953:T:C | rs10509758 | 0.170 | 0.002648 |
| chr10:102931810:A:G | rs79254677 | 0.169 | 0.00274 |
| chr10:102935942:T:G | rs17787717 | 0.160 | 0.003494 |
| chr10:102939221:T:C | rs4919694 | 0.165 | 0.002807 |
| chr10:102939949:C:G | rs139976905 | 0.140 | 0.041704 |
| chr10:102942656:A:C | rs12242000 | 0.166 | 0.003564 |
| chr10:102955774:A:G | rs144350940 | 0.168 | 0.002327 |
| chr10:102957014:A:T | rs113541728 | 0.170 | 0.004697 |
| chr10:102959533:G:A | rs76892505 | 0.166 | 0.004472 |
| chr10:102961964:CTT:C | rs764945861 | 0.169 | 0.0075 |
| chr10:102967767:G:T | rs12258949 | 0.168 | 0.016845 |
| chr10:102985417:T:A | rs11191490 | 0.173 | 0.002669 |
| chr10:102986711:C:T | rs113375453 | 0.172 | 0.002669 |
| chr10:102987837:C:T | rs75219158 | 0.169 | 0.002513 |
| chr10:102993073:T:G | rs4917988 | 0.172 | 0.004515 |
| chr10:102993074:A:T | rs4917379 | 0.172 | 0.004515 |
| chr10:102993969:C:T | rs112240253 | 0.167 | 0.006824 |
| chr10:103043305:C:A | rs12257935 | 0.171 | 0.002847 |
| chr10:103045853:C:A | rs35159404 | 0.171 | 0.002847 |
| chr10:103047127:G:A | rs12266291 | 0.171 | 0.002847 |
| chr10:103049240:G:A | rs12241091 | 0.171 | 0.002847 |
| chr10:103057274:C:T | rs12246689 | 0.172 | 0.002847 |
| chr10:103061499:A:T | rs17727044 | 0.168 | 0.002703 |
| chr10:103062252:C:T | rs77827514 | 0.172 | 0.002847 |
| chr10:103070036:G:A | rs11191545 | 0.172 | 0.003891 |
| chr10:103070877:C:T | rs12264456 | 0.171 | 0.00507 |
| chr10:103073404:C:T | rs12257941 | 0.172 | 0.003891 |
| chr10:103074165:A:G | rs61120766 | 0.172 | 0.003891 |
| chr10:103076344:C:A | rs17727391 | 0.173 | 0.003891 |
| chr10:103078084:G:C* | rs145537350 | 0.143 | 0.024592 |
| chr10:103078753:G:A | rs4917382 | 0.173 | 0.003891 |
| chr10:103082779:G:GTC | rs113304525 | 0.173 | 0.003891 |
| chr10:103167119:G:A | rs112224955 | 0.160 | 0.044737 |
| chr10:103172305:G:A | rs113973959 | 0.160 | 0.044737 |
| chr10:103193264:C:A | rs111622998 | 0.165 | 0.014247 |
| chr10:103210399:C:A | rs111668583 | 0.163 | 0.003933 |
| chr10:103211402:C:T | rs112574306 | 0.159 | 0.012397 |
| chr10:103211446:C:T | rs111750727 | 0.164 | 0.004152 |
| chr10:103218369:T:C | rs113361908 | 0.164 | 0.004152 |
| chr10:103220969:G:T | rs77521106 | 0.159 | 0.012397 |
| chr10:103236502:G:C | rs12253370 | 0.167 | 0.003206 |
| chr10:103243262:G:T | rs78384860 | 0.158 | 0.102714 |
| chr10:103253390:A:C | rs4918001 | 0.163 | 0.03064 |
| chr10:103261717:C:T | rs7903472 | 0.163 | 0.03064 |
| chr10:103274761:C:T | rs112069023 | 0.163 | 0.03064 |
| chr10:103276197:A:G | rs80020194 | 0.158 | 0.102714 |
| chr10:103294707:A:G | rs112991768 | 0.164 | 0.011426 |
| chr10:103298347:A:G | rs75182663 | 0.161 | 0.004652 |
| chr10:103306557:T:C | rs77328741 | 0.161 | 0.004652 |
| chr10:103311080:C:T | rs76695159 | 0.161 | 0.004652 |
| chr10:103315314:T:C | rs117848719 | 0.135 | 0.051913 |
| chr10:103316626:C:T | rs75890466 | 0.156 | 0.004418 |
| chr10:103319373:G:A | rs113555008 | 0.156 | 0.004418 |
| chr10:103353095:C:A | rs117927731 | 0.157 | 0.004418 |
| chr10:103356991:C:A | rs57963697 | 0.161 | 0.004652 |
| chr10:103359203:G:A | rs111968809 | 0.161 | 0.004652 |
| chr10:103396023:G:A | rs17735597 | 0.133 | 0.017493 |

*Indicates SNPs identified in HEALS Confidence Set

**S4B.** SHS Confidence Set 2 based on primary sequencing data

| Variant | rsID | MAF | Posterior Inclusion Probability |
| --- | --- | --- | --- |
| chr10:102882383:CT:C |  | 0.247 | 0.009357 |
| chr10:103213304:A:G | rs10883848 | 0.304 | 0.054785 |
| chr10:103235738:A:G | rs6584542 | 0.306 | 0.168453 |
| chr10:103236031:T:C | rs4917384 | 0.303 | 0.044942 |
| chr10:103236552:G:A | rs10748841 | 0.303 | 0.043365 |
| chr10:103243964:T:G | rs4917385 | 0.303 | 0.044942 |
| chr10:103254113:G:T | rs1712509 | 0.262 | 0.206485 |
| chr10:103263177:T:C | rs1163073 | 0.262 | 0.206485 |
| chr10:103286250:G:A | rs7076274 | 0.259 | 0.036042 |
| chr10:103286932:G:C | rs3740381 | 0.259 | 0.036042 |
| chr10:103295206:C:T | rs7917388 | 0.259 | 0.01203 |
| chr10:103296155:A:G | rs11191642 | 0.259 | 0.01203 |
| chr10:103305216:AC:A | rs1564720985 | 0.258 | 0.01203 |
| chr10:103313313:C:T | rs61869825 | 0.259 | 0.01203 |
| chr10:103315955:C:T | rs12220267 | 0.259 | 0.01203 |
| chr10:103336264:A:G | rs11191658 | 0.259 | 0.01203 |
| chr10:103338267:G:A | rs72846198 | 0.259 | 0.01203 |
| chr10:103343208:G:A | rs11191660 | 0.259 | 0.01203 |
| chr10:103365694:C:A | rs113528138 | 0.260 | 0.01203 |
