# Supplementary material for: Sequencing-based fine-mapping and in silico functional characterization of the 10q24.32 arsenic metabolism efficiency locus across multiple arsenic-exposed populations
Source: PLoS Genet. 2023 Jan 20;19(1):e1010588. doi: 10.1371/journal.pgen.1010588 (PMC9891528; doi:10.1371/journal.pgen.1010588)
Supplement: S5 Table — (DOCX) [file pgen.1010588.s017.docx]

**Table S5** Results of MANTRA meta-analysis of shared signal in HEALS and SHS

| Variant | Direction of Minor Allele Effect in HEALS | Direction of Minor Allele Effect in SHS | Posterior Inclusion Probability |
| --- | --- | --- | --- |
| chr10:103078084* | Decreased DMA% | Decreased DMA% | 0.488 |
| chr10:102875930* | Decreased DMA% | Decreased DMA% | 0.251 |
| chr10:102874717 | Decreased DMA% | Decreased DMA% | 0.076 |
| chr10:102835491 | Decreased DMA% | Decreased DMA% | 0.037 |
| chr10:102831395 | Decreased DMA% | Decreased DMA% | 0.034 |
| chr10:102838165 | Decreased DMA% | Decreased DMA% | 0.032 |
| chr10:102838849 | Decreased DMA% | Decreased DMA% | 0.029 |
| chr10:102827267 | Decreased DMA% | Decreased DMA% | 0.028 |

* SNPs identified as lead signals in association analyses of HEALS and SHS
