# Supplementary material for: Sequencing-based fine-mapping and in silico functional characterization of the 10q24.32 arsenic metabolism efficiency locus across multiple arsenic-exposed populations
Source: PLoS Genet. 2023 Jan 20;19(1):e1010588. doi: 10.1371/journal.pgen.1010588 (PMC9891528; doi:10.1371/journal.pgen.1010588)
Supplement: S6 Table — (DOCX) [file pgen.1010588.s018.docx]

**Table S6.** In-Silico Functional Examination of SHS Primary Sequencing Data Confidence Set 2

| Variant | Coordinates | Candidate cis-Regulatory Element (cCRE) | Transcription Factors binding the cCRE | Histone Marks at this cCRE | Classification |
| --- | --- | --- | --- | --- | --- |
| rs148658224 | chr10:102691571 | None (1 within 2kb) |  |  |  |
| rs4919681 | chr10:102824339 | None |  |  |  |
| rs4917986 | chr10:102870424 | [EH38E1495479](https://screen.wenglab.org/search/?q=chr10%3A102870424&assembly=GRCh38&uuid=3212d256-8e46-4fdf-a75f-361afeb57d28) | 4 | 15 | Proximal enhacer-like signature |
| rs12259506 | chr10:102873533 | None (2 within 2kb) |  |  |  |
| rs10509760 | chr10:102874350 | None (2 within 2kb) |  |  |  |
| rs3740394 | chr10:102874717 | [EH38E1495482](https://screen.wenglab.org/search/?q=rs3740394&assembly=GRCh38&uuid=3212d256-8e46-4fdf-a75f-361afeb57d28) within 2kb | 0 | 4 | Distal enhacer-like signature |
| rs191177668 | chr10:102875930 | [EH38E1495483](https://screen.wenglab.org/search/?q=rs191177668&assembly=GRCh38&uuid=3212d256-8e46-4fdf-a75f-361afeb57d28) within 2kb | 22 | 5 | Distal enhacer-like signature |
| rs111450127 | chr10:102876001 | [EH38E1495483](https://screen.wenglab.org/search/?q=rs111450127&assembly=GRCh38&uuid=3212d256-8e46-4fdf-a75f-361afeb57d28) within 2kb | 22 | 5 | Distal enhacer-like signature |
| rs11191439 | chr10:102878966 | None (1 within 2kb) |  |  |  |
| rs12245779 | chr10:102886187 | None |  |  |  |
| rs12253834 | chr10:102886540 | None |  |  |  |
| rs77505796 | chr10:102888017 | None |  |  |  |
| rs112507051 | chr10:102888709 | None |  |  |  |
| rs17882560 | chr10:102890873 | None |  |  |  |
| rs11191445 | chr10:102891298 | None |  |  |  |
| rs11191446 | chr10:102891723 | None |  |  |  |
| rs76255497 | chr10:102892198 | None |  |  |  |
| rs75691516 | chr10:102892288 | None |  |  |  |
| rs113320965 | chr10:102896558 | None (1 within 2kb) |  |  |  |
| rs80327774 | chr10:102896914 | None (1 within 2kb) |  |  |  |
| rs111638521 | chr10:102898415 | None (2 within 2kb) |  |  |  |
| rs7084472 | chr10:102902240 | None (1 within 2kb) |  |  |  |
| rs12253284 | chr10:102905388 | None (2 within 2kb) |  |  |  |
| rs12261040 | chr10:102905640 | None (2 within 2kb) |  |  |  |
| rs112255065 | chr10:102906131 | None (2 within 2kb) |  |  |  |
| rs112809537 | chr10:102910589 | None (1 within 2kb) |  |  |  |
| rs12251035 | chr10:102911702 | None (2 within 2kb) |  |  |  |
| rs74376228 | chr10:102917905 | None (10 within 2kb) |  |  |  |
| rs10509758 | chr10:102921953 | None (8 within 2kb) |  |  |  |
| rs79254677 | chr10:102931810 | None (1 within 2kb) |  |  |  |
| rs17787717 | chr10:102935942 | None (4 within 2kb) |  |  |  |
| rs4919694 | chr10:102939221 | None |  |  |  |
| rs139976905 | chr10:102939949 | None (1 within 2kb) |  |  |  |
| rs12242000 | chr10:102942656 | None (1 within 2kb) |  |  |  |
| rs144350940 | chr10:102955774 | None |  |  |  |
| rs113541728 | chr10:102957014 | None |  |  |  |
| rs76892505 | chr10:102959533 | [EH38E1495527](https://screen.wenglab.org/search/?q=chr10%3A102959533&assembly=GRCh38&uuid=3212d256-8e46-4fdf-a75f-361afeb57d28) | 16 | 10 | Distal enhacer-like signature |
| rs111595478 | chr10:102961964 | None (5 within 2kb) |  |  |  |
| rs12258949 | chr10:102967767 | None (2 within 2kb) |  |  |  |
| rs11191490 | chr10:102985417 | [EH38E1495550](https://screen.wenglab.org/search/?q=chr10%3A102985417&assembly=GRCh38&uuid=3212d256-8e46-4fdf-a75f-361afeb57d28) | 2 | 17 | Distal enhacer-like signature |
| rs113375453 | chr10:102986711 | [EH38E1495552](https://screen.wenglab.org/search/?q=chr10%3A102986711&assembly=GRCh38&uuid=3212d256-8e46-4fdf-a75f-361afeb57d28) | 6 | 6 | Distal enhacer-like signature |
| rs75219158 | chr10:102987837 | [EH38E1495553](https://screen.wenglab.org/search/?q=chr10%3A102987837&assembly=GRCh38&uuid=3212d256-8e46-4fdf-a75f-361afeb57d28) | 24 | 14 | Distal enhacer-like signature |
| rs4917988 | chr10:102993073 | None (1 within 2kb) |  |  |  |
| rs4917379 | chr10:102993074 | None (1 within 2kb) |  |  |  |
| rs112240253 | chr10:102993969 | None (2 within 2kb) |  |  |  |
| rs12257935 | chr10:103043305 | None (7 within 2kb) |  |  |  |
| rs35159404 | chr10:103045853 | None (4 within 2kb) |  |  |  |
| rs12266291 | chr10:103047127 | None (2 within 2kb) |  |  |  |
| rs12241091 | chr10:103049240 | None (1 within 2kb) |  |  |  |
| rs12246689 | chr10:103057274 | None (3 within 2kb) |  |  |  |
| rs17727044 | chr10:103061499 | None (2 within 2kb) |  |  |  |
| rs77827514 | chr10:103062252 | None (3 within 2kb) |  |  |  |
| rs11191545 | chr10:103070036 | None (6 within 2kb) |  |  |  |
| rs12264456 | chr10:103070877 | None |  |  |  |
| rs12257941 | chr10:103073404 | None (2 within 2kb) |  |  |  |
| rs61120766 | chr10:103074165 | None |  |  |  |
| rs17727391 | chr10:103076344 | None (3 within 2kb) |  |  |  |
| rs145537350 | chr10:103078084 | [EH38E1495605](https://screen.wenglab.org/search/?q=chr10%3A103078084&assembly=GRCh38&uuid=3212d256-8e46-4fdf-a75f-361afeb57d28) | 1 | 5 | Distal enhacer-like signature |
| rs4917382 | chr10:103078753 | None (2 within 2kb) |  |  |  |
| rs541217708 | chr10:103082779 | None (4 within 2kb) |  |  |  |
| rs112224955 | chr10:103167119 | None (3 within 2kb) |  |  |  |
| rs113973959 | chr10:103172305 | None (1 within 2kb) |  |  |  |
| rs111622998 | chr10:103193264 | [EH38E1495690](https://screen.wenglab.org/search/?q=chr10%3A103193264&assembly=GRCh38&uuid=3212d256-8e46-4fdf-a75f-361afeb57d28) | 67 | 28 | Promoter-like signature |
| rs111668583 | chr10:103210399 | None (1 within 2kb) |  |  |  |
| rs112574306 | chr10:103211402 | None (2 within 2kb) |  |  |  |
| rs111750727 | chr10:103211446 | None (2 within 2kb) |  |  |  |
| rs113361908 | chr10:103218369 | None (3 within 2kb) |  |  |  |
| rs77521106 | chr10:103220969 | [EH38E1495715](https://screen.wenglab.org/search/?q=chr10%3A103220969&assembly=GRCh38&uuid=3212d256-8e46-4fdf-a75f-361afeb57d28) | 0 | 3 | DNase |
| rs12253370 | chr10:103236502 | None (1 within 2kb) |  |  |  |
| rs78384860 | chr10:103243262 | None (4 within 2kb) |  |  |  |
| rs4918001 | chr10:103253390 | None (2 within 2kb) |  |  |  |
| rs7903472 | chr10:103261717 | None (1 within 2kb) |  |  |  |
| rs112069023 | chr10:103274761 | None (3 within 2kb) |  |  |  |
| rs80020194 | chr10:103276197 | None (5 within 2kb) |  |  |  |
| rs112991768 | chr10:103294707 | None (1 within 2kb) |  |  |  |
| rs75182663 | chr10:103298347 | None |  |  |  |
| rs77328741 | chr10:103306557 | None (1 within 2kb) |  |  |  |
| rs76695159 | chr10:103311080 | None (1 within 2kb) |  |  |  |
| rs117848719 | chr10:103315314 | None (1 within 2kb) |  |  |  |
| rs75890466 | chr10:103316626 | None (1 within 2kb) |  |  |  |
| rs113555008 | chr10:103319373 | None |  |  |  |
| rs117927731 | chr10:103353095 | None (4 within 2kb) |  |  |  |
| rs57963697 | chr10:103356991 | None (2 within 2kb) |  |  |  |
| rs111968809 | chr10:103359203 | None (2 within 2kb) |  |  |  |
| rs17735597 | chr10:103396023 | [EH38E1495801](https://screen.wenglab.org/search/?q=chr10%3A103396023&assembly=GRCh38&uuid=3212d256-8e46-4fdf-a75f-361afeb57d28) | 51 | 26 | Promoter-like signature |
