# Supplementary material for: Sequencing-based fine-mapping and in silico functional characterization of the 10q24.32 arsenic metabolism efficiency locus across multiple arsenic-exposed populations
Source: PLoS Genet. 2023 Jan 20;19(1):e1010588. doi: 10.1371/journal.pgen.1010588 (PMC9891528; doi:10.1371/journal.pgen.1010588)
Supplement: S7 Table — (DOCX) [file pgen.1010588.s019.docx]

**Table S7** Co-localization of eQTLs for genes in the 10q24.32 region (excluding AS3MT) DMA% association signal (lead SNP rs4919687) identified in Bangladeshi individuals with posterior probability of colocalization > 80% (for p12=5x10-6)

| Gene | Tissue | eQTL Lead SNP | PP of Co-localization Under Different Assumptions | | | | Adjusted to Isolate eQTLs |
| --- | --- | --- | --- | --- | --- | --- | --- |
|  |  |  | 50% of DMA%  SNPs are eQTLs | 25% of DMA%  SNPs are eQTLs | 10% of DMA%  SNPs are eQTLs | 5% of DMA%  SNPs are eQTLs |  |
| **CYP17A1OS** | Thyroid | rs11191401 | 0.96 | 0.89 | 0.73 | 0.57 | 0 SNPs |
| **CYP17A1** | Brain Frontal Cortex | rs743575 | 0.92 | 0.79 | 0.56 | 0.38 | 0 SNPs |
| **BORCS7** | Brain Anterior Cingulate Cortex | rs11191421 | 0.82 | 0.61 | 0.34 | 0.20 | 0 SNPs |
|  | Brain Caudate Basal  Ganglia |  | 0.89 | 0.73 | 0.48 | 0.31 | 0 SNPs |
|  | Brain Hypothalamus |  | 0.94 | 0.85 | 0.66 | 0.48 | 0 SNPs |
|  | Brain Substantia Nigra |  | 0.90 | 0.76 | 0.51 | 0.33 | 0 SNPs |
|  | Minor Salivary  Gland |  | 0.91 | 0.78 | 0.54 | 0.36 | 0 SNPs |
|  | Nerve Tibial |  | 0.84 | 0.65 | 0.39 | 0.23 | 0 SNPs |
|  | Pituitary |  | 0.95 | 0.88 | 0.71 | 0.53 | 0 SNPs |
|  | Small Intestine Terminal Ileum |  | 0.96 | 0.88 | 0.72 | 0.55 | 0 SNPs |
|  | Stomach |  | 0.96 | 0.90 | 0.75 | 0.58 | 0 SNPs |
|  | Adrenal Gland |  | 0.97 | 0.91 | 0.77 | 0.61 | 2º SNP |
|  | Brain Amygdala |  | 0.94 | 0.85 | 0.66 | 0.48 | 2º SNP |
|  | Colon Sigmoid |  | 0.81 | 0.60 | 0.34 | 0.20 | 2º SNP |
|  | Heart Atrial Appendage |  | 0.89 | 0.74 | 0.49 | 0.32 | 2º SNP |
|  | Liver |  | 0.97 | 0.91 | 0.78 | 0.62 | 2º SNP |
|  | Lung |  | 0.89 | 0.74 | 0.49 | 0.31 | 2º SNP |
|  | Whole Blood |  | 0.97 | 0.92 | 0.79 | 0.64 | 2º SNP |
|  | Brain Cerebellum | rs4919690 | 0.98 | 0.95 | 0.86 | 0.75 | 0 SNPs |
|  | Prostate |  | 0.98 | 0.94 | 0.84 | 0.71 | 0 SNPs |
|  | Spleen |  | 0.98 | 0.95 | 0.86 | 0.75 | 0 SNPs |
|  | Adipose Visceral Omentum |  | 0.99 | 0.97 | 0.91 | 0.83 | 2º SNP |
|  | Brain Hippocampus |  | 0.97 | 0.93 | 0.82 | 0.68 | 2º SNP |
|  | Colon Transverse |  | 0.99 | 0.98 | 0.94 | 0.88 | 2º SNP |
|  | Esophagus  Gastroesophageal Junction |  | 0.99 | 0.98 | 0.93 | 0.87 | 2º SNP |
|  | Pancreas |  | 0.99 | 0.98 | 0.94 | 0.88 | 2º SNP |
|  | Artery Aorta |  | 0.99 | 0.98 | 0.94 | 0.88 | 2º,3ºSNP |
