# Supplementary material for: Sequencing-based fine-mapping and in silico functional characterization of the 10q24.32 arsenic metabolism efficiency locus across multiple arsenic-exposed populations
Source: PLoS Genet. 2023 Jan 20;19(1):e1010588. doi: 10.1371/journal.pgen.1010588 (PMC9891528; doi:10.1371/journal.pgen.1010588)
Supplement: S8 Table — (DOCX) [file pgen.1010588.s020.docx]

**Table S8** Co-localization (PP>80%) of mQTLs in the 10q24.32 region identified in GTEx tissues with the association signal for DMA% (lead SNP rs4919687) identified in a Bangladeshi population across a range of prior probabilities

| Tissue | CpG | mQTL  Lead SNP | PP of Co-localization Under Different Assumptions | | | |
| --- | --- | --- | --- | --- | --- | --- |
|  |  |  | 50% of DMA%  SNPs are mQTLs | 25% of DMA%  SNPs are mQTLs | 10% of DMA%  SNPs are mQTLs | 5% of DMA%  SNPs are mQTLs |
| Colon Transverse | cg17932736 | rs11191421 | 0.916 | 0.791 | 0.562 | 0.379 |
| Ovary | cg02786313 | rs12775431 | 0.912 | 0.784 | 0.55 | 0.368 |
| Ovary | cg15744005 | rs4919690 | 0.871 | 0.702 | 0.443 | 0.274 |
