# Supplementary material for: Sequencing-based fine-mapping and in silico functional characterization of the 10q24.32 arsenic metabolism efficiency locus across multiple arsenic-exposed populations
Source: PLoS Genet. 2023 Jan 20;19(1):e1010588. doi: 10.1371/journal.pgen.1010588 (PMC9891528; doi:10.1371/journal.pgen.1010588)
Supplement: S9 Table — (DOCX) [file pgen.1010588.s021.docx]

**Table S9** Effect modification analysis of the effect of the interaction between DMA% associated variants and sex on DMA% in HEALS

| **Variables** | **β** | **S.E** | **P-Value** |
| --- | --- | --- | --- |
| **rs145537350** | -14.02 | 2.01 | 3.54x10^-12^ |
| Female | 2.6 | 0.35 | 8.72x10^-14^ |
| rs145537350*Female | 3.04 | 3.1 | 0.327 |
| **rs12573221** | 2.76 | 0.52 | 1.01x10^-7^ |
| Female | 2.62 | 0.40 | 9.45x10^-11^ |
| rs12573221*Female | 0.34 | 0.81 | 0.68 |
| **rs4919687** | -3.43 | 0.53 | 1.74x10^-10^ |
| Female | 2.51 | 0.4 | 3.52x10^-10^ |
| rs4919687*Female | 0.68 | 0.83 | 0.41 |

Abbreviations: OR, odds ratio; S.E, standard error
