# Supplementary material for: Sequencing-based fine-mapping and in silico functional characterization of the 10q24.32 arsenic metabolism efficiency locus across multiple arsenic-exposed populations
Source: PLoS Genet. 2023 Jan 20;19(1):e1010588. doi: 10.1371/journal.pgen.1010588 (PMC9891528; doi:10.1371/journal.pgen.1010588)
Supplement: S10 Table — (DOCX) [file pgen.1010588.s022.docx]

**Table S10** Gene-Environment analysis of the effect of the interaction between DMA% associated variants and water arsenic concentration on DMA%

| **Variables** | **β** | **S.E** | **P-Value** |
| --- | --- | --- | --- |
| **rs145537350** | -11.26 | 2.85 | 7.8x10^-5^ |
| Medium Arsenic Exposure | -1.55 | 0.42 | 2.7x10^-4^ |
| High Arsenic Exposure | -2.64 | 0.42 | 5.9x10^-10^ |
| rs145537350*Medium Exposure | -0.4 | 3.7 | 0.91 |
| rs145537350*High Exposure | -4.4 | 4.03 | 0.27 |
| **rs12573221** | 2.44 | 0.72 | 7.35x10^-4^ |
| Medium Arsenic Exposure | -2.01 | 0.49 | 3.69x10^-5^ |
| High Arsenic Exposure | -2.9 | 0.49 | 2.84x10^-9^ |
| rs12573221*Medium Exposure | 1.05 | 0.99 | 0.29 |
| rs12573221*High Exposure | 0.44 | 0.999 | 0.66 |
| **rs4919687** | -2.36 | 0.71 | 8.96x10^-4^ |
| Medium Arsenic Exposure | -1.5 | 0.48 | 1.71x10^-3^ |
| High Arsenic Exposure | -2.29 | 0.49 | 2.5x10^-6^ |
| rs4919687*Medium Exposure | -0.83 | 1.03 | 0.42 |
| rs4919687*High Exposure | -1.43 | -/99 | 0.15 |

Abbreviations: OR, odds ratio; S.E, standard error
