# Supplementary material for: Sequencing-based fine-mapping and in silico functional characterization of the 10q24.32 arsenic metabolism efficiency locus across multiple arsenic-exposed populations
Source: PLoS Genet. 2023 Jan 20;19(1):e1010588. doi: 10.1371/journal.pgen.1010588 (PMC9891528; doi:10.1371/journal.pgen.1010588)
Supplement: Attachment — Submitted filename: Formal Response to Review Final2.docx [file pgen.1010588.s027.docx]

**Review Comments & Formal Response to Comments**

**PLOS Genetics**

**Reviewer 1:**

1. I would urge the authors to use caution with the use of terms such as “causal variants” and “functional characterization.” The causal variants were identified in relation to expression (not phenotype) and the functional characterization was carried out in silico and not using in vitro or in vivo techniques. It is strongly suggested to add “in silico” prior to the use of functional characterization throughout the manuscript including in the title to prevent from misleading the reader.

We have now added the term “in silico” to the title of the paper and to the title of Fig. 5. Additionally, we have toned down the language surrounding causality in both the abstract and the author summary. Finally, throughout the paper, we have ensured that all causal language was qualified with terms like “potential” and “candidate” outside of specific method descriptions in order to ensure that we do not overstate claims of causality.

2. A description of other factors that may influence the differences in the finding of AME-associated SNPs/variants across studies is needed. The authors have done a good job listing some of the factors that could have influenced the finding of different AME-associated variants across populations. Please do expand this section to describe the following: (1) how differences in arsenic exposure levels might have influenced the results (e.g., very high exposure to low exposure), (2) how differences in sample sizes of the populations may have influenced the results (e.g., HEALS >1000, NH<250), and (3) how differences in subject recruitment and inclusion may have influenced the results (e.g. the NH study recruited those w/w/o skin cancer).

For point (1), we now include additional details on our gene-environment interaction analyses to the discussion section, describing the way in which population differences in arsenic exposure levels may impact our findings. We have added Table S10, showing the results of this analysis HEALS in which we found no evidence of effect modification between arsenic exposure and our identified variants. We also performed this analysis in the NHSCS, but due to the narrow exposure in this population the results are not included in the table. However, NHSCS results agree with those in HEALS and no interaction was found.

The Discussion section of manuscript now states, “Gene-gene or gene-environment interactions could result in different effect size estimates across populations (34,36). Differences in exposure level across populations may also impact the observed associations (36,37,39). For instance, attenuation of SNP effects in populations with low exposure may result in low power to detect association(36). We examined the gene-environment interaction between our lead SNPs and arsenic exposure in HEALS and while arsenic exposure did have an independent effect on DMA%, there was no evidence of effect modification between any of our identified variants and arsenic exposure (Supplemental Table S10).”

For point (2), we added an explicit discussion of sample size to the paragraph describing the impact of MAF on our ability to detect shared causal variants. We also discussed population size in the context of co-localization limitations.

In the Discussion we now state, “Our study used different sample sizes for our three populations. Larger sample sizes, as seen in HEALS, increase the study power and decrease the standard error of effect estimates, and thus improve our ability to identify association signals (40). It is possible that we simply lacked the power to identify all association signals across all populations.” Additionally, we state, “The population mismatch between our studies of AME (Bangladeshi and American Indian populations) and GTEx donors (primarily European ancestry) likely decreased our power to detect co-localization in both our eQTL and mQTL analysis.”

For point 3, we added a paragraph discussing differences in subject recruitment and inclusion and its potential impact on the results of the study to the Discussion section of the manuscript.

We now state, “In addition to these factors, differences in subject recruitment and inclusion among the studies used for this work may also have continued to the differences in association signals. NHSCS included individuals both with and without skin cancer. Arsenic exposure and AME are risk factors for skin cancer, so this selection could contribute to biases in observed associations (27,42).”

3. The authors examine AS3MT expression across tissues, but do not provide a differential analysis of the baseline expression in the tissues. This may impact the biological relevance of the findings. AS3MT has highly variable expression across tissues. The authors should provide the context for the baseline expression in these tissue and detail how these results impacted by potentially very low levels of AS3MT in various tissues.

We now explicitly mention that *AS3MT* has highly variable expression across tissues and provide the range of expression near the reference to Supplemental Figure S10 that shows baseline *AS3MT* expression across tissues. This is found in the Results section *cis-eQTL identification and co-localization analysis.*

In the Discussion, we contextualize our results given the tissue expression variation. “*AS3MT* is expressed in most tissues at detectable levels (Fig.S10), allowing us to examine co-localization across a wide range of tissues. Expression is highest in the adrenal gland, potentially due to co-regulation with nearby CYP17A1 which plays a role in steroid hormone formation or for protection against arsenic which can disrupt endocrine function (43,44). While co-localization was not detected for adrenal tissue, it was detected in many tissue types with low expression (e.g. subcutaneous adipose). This suggests that our results were not driven by the variability in expression across tissues and that low expression levels did not prevent QTL detection.”

4. The authors include the covariates of age and sex in their primary models. Please detail how these were selected for inclusion. That these factors are associated with AME is not in question. Rather, please provide support for these factors being associated with AS3MT genotype.

Clarification is now provided in the Methods section that these two covariates are associated with AME and that adjusting for them in our models removed variation in the outcome and therefore may increase our study’s power to detect association. These covariates were not included to adjust for potential confounding.

In the *Association Analysis* section of the methods we now state: “Both sex and age are associated with AME; therefore, they were included as covariates in order remove variation in the outcome of interest and increase the power of our analysis.”

5. Related to the comment above, the cohorts vary significantly in BMI. How would the results change if obesity had been included in the models?

In Methods, we clarified that we never analyze data across cohorts; rather, each cohort is analyzed individually and then the results are compared. Within each cohort, we did check to see if the addition categorical BMI variable altered the significance of our lead SNPs. In the Results, we now describe this analysis: “We examined the impact of BMI as a covariate in each cohort. While BMI is associated with DMA% in each cohort, the association for the lead SNPs identified in each cohort are largely unchanged.”

6. Please provide the beta estimate of rs191177668 in relation to AME in HEALS.

We added this information to the results section in the discussion of the observed association signals. “The shared lead SNP in SHS and NHSCS, rs191177668, has a p-value of 8x10-8 and beta estimate of -0.062 in HEALS (isolated primary signal). Controlling for the primary signal, this SNP has p-values of 0.6 and 0.2 in the HEALS secondary and tertiary analyses.”

7. Please add the liver to Figure 6.

The liver was not included in this figure due to the lack of co-localization identified in the analysis of this tissue type. However, we have now added the liver as a supplemental figure (Fig. S11) in the manuscript.

8. The eQTL analysis reads as if it is a biologically separate analysis from the identification of AME-associated SNPs. The authors should continue the information flowing from the analysis previously to add the estimates of association with gene expression for the identified AME-associated SNP (e.g., what was the estimate of rs191177668)

We acknowledge that these analyses are separate. We chose to perform QTL discovery first (followed by colocalization analysis) because we believe that focusing on AME-associated SNPs as candidate eQTL SNPs is a less rigorous approach. Showing evidence of co-localization is critical to making the argument that an association observed in GWAS may be due to an eQTL (i.e., due to a common causal variant).

With that in mind, we have now added a transition sentence to the cis-eQTL Results section that improves the flow between the AME results and the QTL results. We now state, “Following identification of these QTLs, we wanted to determine if any eQTLs shared a common causal variant with our AME association signals thereby representing a regulatory mechanism by which our identified SNPs impact AME.”

We have also added a transition sentence to our co-localization results section, “Following the identification of eQTLs in high LD with our AME-associated SNPs, we performed a co-localization analysis to determine whether the same causal variants impact AME and AS3MT expression, providing a regulatory mechanism by which our identified SNPs influence AME.”

We have also added the following sentence on our AME SNPs to the QTL results section “Examining the individual lead SNPs associated with urinary DMA% in the context of our eQTL analysis, we found that HEALS lead SNP rs4919687 had p < 5x10-8 in forty tissue types and the shared lead SNP in SHS and NHSCS, rs191177668, had p-value < 5x10-8 in eight tissue types (based on primary eQTL analyses). The second lead SNP in SHS (rs4919688) was not identified in any of our QTL analyses.”

9. Additional citations are needed. Specifically, the authors state “In the cohorts with available measurements of arsenic in drinking water (HEALS and NHSCS) water arsenic exposure was inversely associated with DMA% (Fig S8), as reported previously for HEALS”. Please add the citation for where this was previously reported.

We have added the citations (Ahsan, 2007 and Jansen 2016) which is where this was previously reported.

10. Needed text additions. In the abstract, it states that “There was one signal shared across all three examined populations.” But the exciting signal is not detailed. Realizing that this is complicated by the fact the one SNPs is shared between the SHS and NH cohorts but not directly in HEALS, it might make sense to list the common RS as well as the LD-associated RS in HEALS. Please add more information in the abstract stating which signal this is.

The signal we were referring to was the one SNP shared between SHS and NHSCS which was in LD with the signal in HEALS. We now clarify this language in the abstract, stating: “There was one signal shared across all three populations, represented by the same SNP in American Indians and European Americans (rs191177668) that was in strong linkage disequilibrium (LD) with a lead SNP in Bangladesh (rs145537350).”

11. Also in the abstract, the authors state “causal variant across populations, and elucidate potential biological mechanisms” but do not state a mechanism for what? This would be improved by adding additional text to the end of this sentence

Additional text was added to the end of this sentence in the abstract “Taken together, our analyses detect multiple potential causal variants in the 10q24.32 region influencing AME, including a variant shared across populations, and elucidate potential biological mechanisms underlying the impact of genetic variation on AME”

12. The authors state the arsenic metabolism is “believed” to occur primarily in the liver. Why do they use this qualifier?

We have removed this language. We did not want to overstate the role of the liver as arsenic metabolism may occur in other tissues including the kidney and erythrocytes. However, we have now clarified this with additional citations and modified the language. The text now states “iAs metabolism in humans is composed of a series of reduction and methylation reactions occurring primarily in the liver with some metabolism potentially occurring in other tissues such as the kidney (20,21)”

13. Suggested wording modification from “is comprised of” to is composed of, or comprises.

We have modified the language as suggested, and the word “composed” is now used instead of “is comprised of.”

14. The authors note “Prior studies have found that higher levels of MMA% and/or lower levels of DMA% are associated with increased risk for.” Here and throughout make sure to add the term urinary, as of course the proportions of these metabolites vary on tissue where liver, or target tissues versus urine would be very different.

The word urinary is now included both in this specific place and in several additional locations throughout the manuscript to ensure that this was clarified.

15. No prior evidence suggests a role for BORCS7 in arsenic metabolism; …what about mirek’s paper

We have now added a reference to the 2020 paper “Arsenic Metabolism in Mice Carrying a BORCS7/AS3MT Locus Humanized by Syntenic Replacement”. In this paper, the correlation of AS3MT and BORCS7 are discussed, but no specific role for BORCS7 in arsenic metabolism (separate from AS3MT) was mentioned. This information is now included in the Discussion section of the manuscript text.

The Discussion now states, “The correlated expression of the two genes has been noted previously (46). A mouse strain carrying a human *BORCS7/AS3MT* was created to study arsenic metabolism as the *AS3MT* promoter abuts the 3’ UTR of *BORCS7*(47). However, this study did not describe any specific role for *BORCS7* in arsenic metabolism. It is likely that the SNPs in this region are pleiotropic, influencing both *AS3MT* and the expression of the surrounding genes.”

**Reviewer 2:**

1) It is very difficult to identify causal variants with low-to-moderate effect sizes across different populations when there are other factors that influence the phenotype of interest, namely the arsenic metabolism efficiency (AME). I have some comments on how the authors have handled this. According to table 1, there are individuals with a very high fraction of DMA in urine, i.e. >85%. It is very likely that among those individuals, some of the DMA does not come from inorganic arsenic, but from the metabolism of organic arsenic present in food stuff. Including those individuals in the SNP-AME analysis will blur the associations. The authors try to take this into account by adjusting for arsenobetaine, but there may still be residual confounding since the DMA and arsenobetaine are correlated in all 3 cohorts (no correlation coefficient is presented). I therefore suggest that you do a sensitivity analysis by excluding individuals with an arsenic metabolite pattern (>85%) that does not reflect the activity of AS3MT.

We now explicitly include the strength of correlation (r) between DMA% and arsenobetaine in the 3 cohorts in the *Sensitivity Analysis* section of the results. We state: “Arsenobetaine, a form of organic arsenic found in seafood (see methods) was correlated with DMA% in all three cohorts (correlation of 0.18, 0.12, and 022 in HEALS, SHS, and NHSCS respectively); however, no clear differences were seen between our initial analysis results and those from a model that included adjustment for arsenobetaine.”

We performed the suggested sensitivity analysis and include information regarding the number of individuals excluded from the analysis due to the DMA% cut-off of 85%. In the Sensitivity Analysis section of the Results we state, “To further confirm our results, we performed a sensitivity analysis, excluding individuals with DMA% >85 (76 in HEALS, 224 in NHSCS, and 169 in SHS) to avoid the inclusion of DMA measures strongly influenced by exposure to organic arsenic. Here, we found no significant change in results with our lead SNPs still appearing among the top SNPs in the association analyses of all three populations.”

We also now cite work regarding rice as a source of arsenic exposure in HEALS. The introduction section now states, “Other sources of arsenic exposure include the consumption of contaminated seafood and rice (20–22).” Additionally, in the Discussion section of the paper we acknowledge the limitation of our study due to the lack of measurements of organic arsenic sources. We state: “Additionally, in the populations examined for this study, we are not able to fully assess the contribution of organic sources of arsenic to variability in our AME phenotype, which could potentially bias the associations observed.”

We also add more information to the Methods section, *Association Analysis,* regarding arsenobetaine. “Arsenobetaine is an organic form of arsenic that is highly stable and the major arsenic species found in most seafood(73). Arsenobetaine is a marker of seafood intake, a diet that can also contain arsenosugars and arsenolipids, which were not measured but which have the potential to contribute to DMA%(73). We therefore examined the relationship between arsenobetaine and DMA% and adjusted for arsenobetaine in multivariate models.”

2) Both sex and smoking (smokers and men have a lower AME), and in some populations also the arsenic exposure level, affect the AME. It is not clear how smoking is handled in the association analyses. In table 1 it says “has smoked” - does this mean that all study participants were non- or ex-smokers? If not, did you do a sensitivity analysis excluding the current smokers? Further, did you stratify the analysis for men and women (you state that “the effects of our identified variants were similar in males and females”)? And did you also try to adjust for total sum of inorganic arsenic metabolites as you state that is influences the AME in some of the cohorts?

Our participants included both current and former smokers. We performed a sensitivity analysis excluding smokers in NHSCS, we find that the lead SNP remains among the top SNPs (second smallest P-value). We performed a series of interaction analyses in which a categorical smoking variable (current, former, and never smoker) was included in a linear model with the identified lead SNPs to examine both the main effect and interaction effects of these variables on DMA%. Overall, we found no evidence that the effects of our identified SNPs vary by smoking in any of the three cohorts. We have added this information to the *Effect Modification* *Analysis* section of the Results. We state: “Finally, we examined the interaction between smoking and DMA% and observed evidence of interaction between current smoking and the lead SNP in NHSCS (p=0.04), but not in HEALS or SHS.”

Additionally, we now include the results of an interaction analysis to dive further into the potential impact of sex on our results within the Results *Effect Modification Analysis* section and include these results in the manuscript as a supplemental table (S9). We find both sex and genetics are significant, but we see not significant interaction between sex and any of the lead SNPs in HEALS, and state: “We found no interaction between sex and the genetic effects on DMA% (Supplemental Table 9); the effects of our identified variants were similar in males and females.”

We cannot adjust for the sum of arsenic species in our model because it is the denominator of our outcome measure. In our calculation of the DMA% variable, we have already adjusted for the total. This is why we did not include total as a covariate in the analysis. In terms of the inclusion of total water arsenic in the model as a proxy for exposure, we include the GxE results in the paper whose results show that both water arsenic levels and genetics are significantly associated with DMA% however we observe no effect modification/interaction between the exposure and the lead SNPs. In the Discussion section we now state: “Differences in exposure level across populations may also impact the observed associations (39,40,42). For instance, attenuation of SNP effects in populations with low exposure may result in low power to detect association (39). We examined the gene-environment interaction between our lead SNPs and arsenic exposure in HEALS and while arsenic exposure did have an independent effect on DMA%, there was no but did not find evidence of effect modification between any of our identified variants and arsenic exposure (Supplemental Table S10).”

3) What is novel with the study should be better explained. The findings that AS3MT genotype influences DNA methylation and in turn gene expression of multiple genes in the AS3MT region has been known for a decade and this should be better acknowledged in the discussion. Further, it should be elaborated on how causal variant(s) can be identified further.

We have added clarification to the Discussion section: “The relationships among *AS3MT* genotype, gene expression in the 10q24.32 region, and arsenic metabolism are well-established; however, questions remain regarding the precise causal variants driving these associations, the potential differences in associations across ancestry groups, as well as the specific genes in the region influenced by 10q24.32 genotypes across different tissue types.”

We also further clarify the novelty of our population-specific findings in the Discussion section, “A novel finding of our analysis is the identification of associations that appear to be population-specific in both HEALS and SHS, which require further examination." We include further discussion of the way in which our study builds on previous work is in the final paragraph of the discussion and added a sentence describing potential future work for the establishment of causal variants: “Future studies can build on these findings, potentially establishing cellular or animal models with perturbations of potential causal sites in order to directly assess the impact of specific alleles on arsenic metabolism.”

4) De Loma et al (2022) recently identified potentially functional SNPs in the AS3MT region linked to AME in South American populations. Are these polymorphisms in LD with the key ones identified here?

We have added the following text to discussion section: “Previous studies have found signs of positive selection near the AS3MT gene associated with efficient arsenic metabolism (56). They found selection signals in multiple populations including several from South America and East Asia and found that several the SNPs associated with the protective haplotype were also associated with MMA%. None of the identified SNPs showed a significant association with DMA%, though in the context of our study one SNP did appear in LD with the secondary lead SNP in SHS.”

5) It is striking that AS3MT is expressed in so many tissues not primarily involved in the arsenic detoxification (e.g. the cardiovascular system and the brain). Moreover, you find multiple SNPs linked to %DMA as well as eQTL and mQTLs in different tissues, but few in the liver (this may reflect a database bias though). How do you explain this? Could it be that the SNPs are pleiotropic, both influencing AME and surrounding gene expression and in turn other pathways? And if so, how can you explain this co-regulation of genes with very different function, maintained by strong LD in the 10q32 region?

We have added more details regarding the liver analyses and our results to the manuscript. We clarify that we do observe evidence of an eQTLin the liver (GTEx), but the signal is not especially strong given the limited sample size for liver eQTL analyses. Evidence of co-localization is suggestive, but not strong across all priors. Therefore, we added the following text to the Discussion section of paper: “The liver is the major site of arsenic metabolism, but we observed only suggestive evidence of co-localization in this tissue type (Table 5, Fig. S11). We detected *AS3MT* eQTLs in liver (rs4919690, p=7.15x10-8), but our relatively small sample size may have limited our statistical power to robustly identify co-localization across all sets of priors analyzed.”

Pleiotropy is possible and we know there is co-regulation. Our data also suggests co-regulation in some tissue types, and we have added the following text to the paper in *BORCS7* section of the Discussion: “The correlated expression of the two genes has been noted previously (49). A mouse strain carrying a human *BORCS7/AS3MT* was created to study arsenic metabolism as the *AS3MT* promoter abuts the 3’ UTR of *BORCS7*(50). However, this study did not describe any specific role for *BORCS7* in arsenic metabolism. It is likely that the SNPs in this region are pleiotropic, influencing both AS3MT and the expression of the surrounding genes.”

6) Introduction: Do not write AS3MT in italics when you refer to the protein.

We have corrected this issue in the introduction of the manuscript.
